# Respiratory and metabolic acidosis correction with the ADVanced Organ Support system

**DOI:** 10.1186/s40635-019-0269-7

**Published:** 2019-09-18

**Authors:** Aritz Perez Ruiz de Garibay, John A. Kellum, Johannes Honigschnabel, Bernhard Kreymann

**Affiliations:** 1 ADVITOS GmbH, Agnes-Pockels-Bogen 1, 80992 Munich, Germany; 2 Center for Critical Care Nephrology, Department of Critical Care Medicine, University of Pittsburgh Medical Center, Pittsburgh, USA; 3 Faculty of Medicine, Ludwig-Maximilians-Universität München, Munich, Germany

**Keywords:** Multiple organ failure, Respiratory hemodialysis, Extracorporeal carbon dioxide removal, Respiratory acidosis, Metabolic acidosis, Lactic acidosis, ECCO2R, Albumin dialysis, Extracorporeal organ support

## Abstract

**Background:**

The lung, the kidney, and the liver are major regulators of acid-base balance. Acidosis due to the dysfunction of one or more organs can increase mortality, especially in critically ill patients. Supporting compensation by increasing ventilation or infusing bicarbonate is often ineffective. Therefore, direct removal of acid may represent a novel therapeutic approach. This can be achieved with the ADVanced Organ Support (ADVOS) system, an enhanced renal support therapy based on albumin dialysis. Here, we demonstrate proof of concept for this technology.

**Methods:**

An ex vivo model of either hypercapnic (i.e., continuous CO_2_ supply) or lactic acidosis (i.e., lactic acid infusion) using porcine blood was subjected to hemodialysis with ADVOS. A variety of operational parameters including blood and dialysate flows, different dialysate pH settings, and acid and base concentrate compositions were tested. Comparisons with standard continuous veno-venous hemofiltration (CVVH) using high bicarbonate substitution fluid and continuous veno-venous hemodialysis (CVVHD) were also performed.

**Results:**

Sixty-one milliliters per minute (2.7 mmol/min) of CO_2_ was removed using a blood flow of 400 ml/min and a dialysate pH of 10 without altering blood pCO_2_ and HCO_3_^−^ (36 mmHg and 20 mmol/l, respectively). Up to 142 ml/min (6.3 mmol/min) of CO_2_ was eliminated if elevated pCO_2_ (117 mmHg) and HCO_3_^−^ (63 mmol/l) were allowed. During continuous lactic acid infusion, an acid load of up to 3 mmol/min was compensated. When acidosis was triggered, ADVOS multi normalized pH and bicarbonate levels within 1 h, while neither CVVH nor CVVHD could. The major determinants to correct blood pH were blood flow, dialysate composition, and initial acid-base status.

**Conclusions:**

In conclusion, ADVOS was able to remove more than 50% of the amount of CO_2_ typically produced by an adult human. Blood pH was maintained stable within the physiological range through compensation of a metabolic acid load by albumin dialysate. These in vitro results will require confirmation in patients.

## Introduction

Lung, kidney, and hepatic dysfunction are common in the critically ill, and acid-base regulation is frequently compromised in these patients. Acidosis is commonly associated with high mortality rates in critically ill and injured patients. Recently, a strong correlation was found between hypercapnic acidosis and increased hospital mortality in mechanically ventilated patients, compared to compensated hypercapnia or normocapnia [1]. Indeed, a delayed pH normalization is associated with increased mortality in the intensive care unit (ICU), reaching 57% in cases of severe metabolic or mixed acidemia [2]. If accompanied by hyperlactatemia, these values could go above 80% [3, 4].

While acidosis is well tolerated in healthy humans, acidosis leads to a myriad of physiologic effects that can be deleterious and thus contribute to morbidity and mortality in patients [5]. In fact, an interaction exists between acidosis and inflammation, which is specifically relevant in critically ill patients [6, 7]. Acidosis can impair several immune response mechanisms, including lymphocyte cytotoxicity, complement activation, or antibody binding to leukocytes [8]. Since some of these findings have been already reported in patients [7], acid-base imbalances should be considered in the context of a host response to an aggression, and not as an isolated insult. Moreover, pH might also influence normal physiology, among others, modulating oxygen affinity to hemoglobin [9, 10], promoting vasoconstriction in the lungs [11], altering potassium and calcium levels, reducing glomerular filtration rate [12], reducing intestinal mobility [13], impairing coagulation [14], or depressing myocardial contractility [9].

**Fig. 1:**
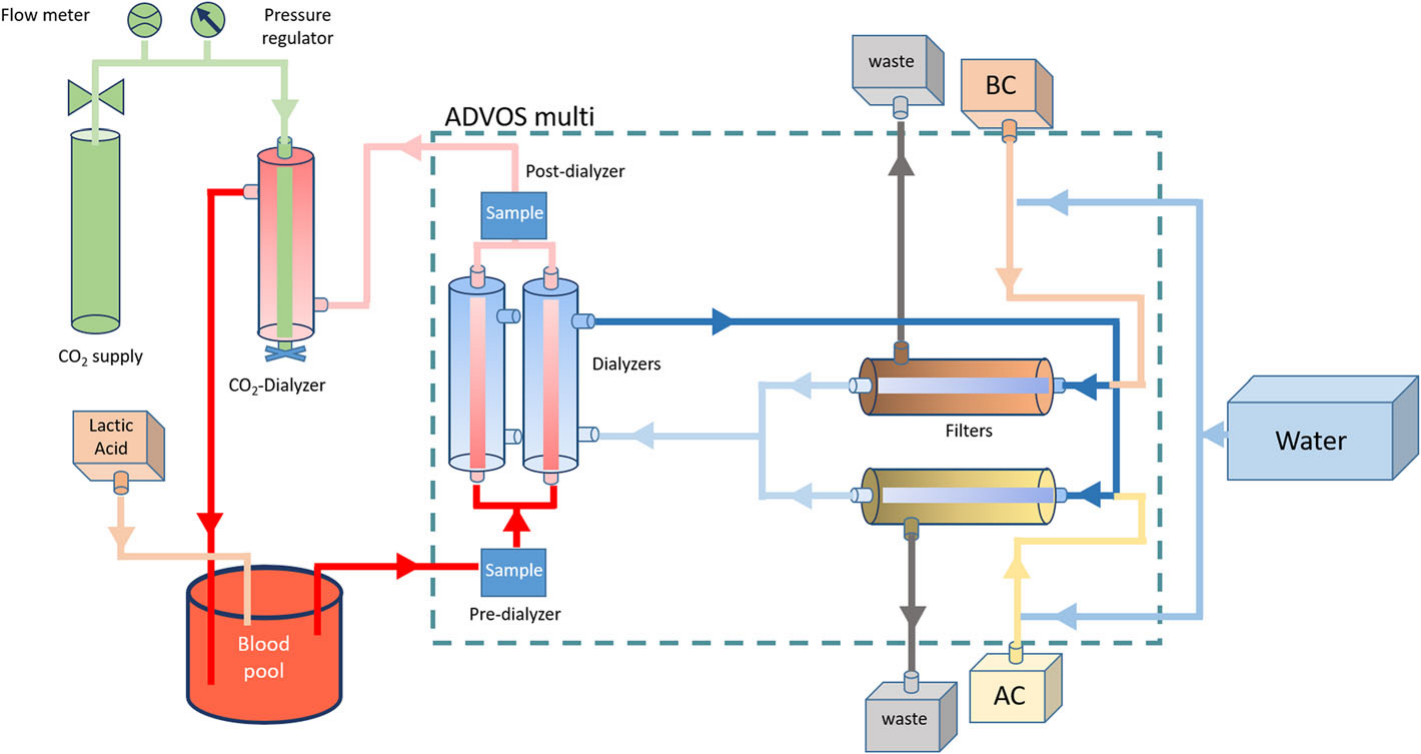
Schematic representation of ADVOS multi device with a continuous CO_2_ supply. Lactic acid was supplied in blood only for experimental settings 2 and 4 (see Table 1 for more details)

The ADVanced Organ Support (ADVOS) system (ADVITOS GmbH, Munich, Germany—previously, Hepa Wash GmbH) is an albumin-based hemodialysis device initially designed to support the liver and kidney of ICU patients. As a hemodialysis system, it removes water-soluble substances, while the albumin dialysate allows to remove protein-bound toxins too [15–17]. The ADVOS system consists of three circuits: an extracorporeal blood, a dialysate, and an ADVOS multi circuit with an acidic and an alkaline path (Fig. 1). Briefly, the dialysate’s albumin binds protein-bound substances that diffuse from blood into the dialysate through a semi-permeable membrane in the extracorporeal circuit. Differently to conventional single pass albumin dialysis (SPAD), in the ADVOS system, the dialysate is not systematically discarded and recirculates then through the dialysate circuit into the ADVOS multi circuit. There, dialysate albumin is recycled by systematically modifying the tertiary structure of albumin through temperature and pH changes. This facilitates the release of toxins from albumin (cationic—e.g., copper—and anionic—e.g., bilirubin—substances in the acidic and alkaline paths, respectively) and makes it ready for further binding. pH changes are possible by the addition of an acidic and an alkaline concentrate, whose customizable mixing relation forms a dialysate with a variable composition. This includes modifiable carbonate, sodium, or chloride concentrations that allow to achieve a customizable dialysate with a pH from 7.2 to 10.0. Consequently, this latter feature enables a continuous control and adjustment of dialysate acid-base properties, which in turn corrects deviations in blood pH (e.g., acidosis) by means of altering blood pCO_2_, strong ion difference, or both [18].

In the present study, the ability of ADVOS to eliminate CO_2_ and to correct blood pH was investigated in an ex vivo model of either lactic or hypercapnic acidosis, using porcine blood and a continuous supply of lactic acid and/or CO_2_, respectively. A variety of operational parameters, including blood and dialysate flows as well as different dialysate pH settings and acid and base concentrate compositions, were tested. In addition, comparisons with standard continuous veno-venous hemofiltration (CVVH) using high bicarbonate substitution fluid and continuous veno-venous hemodialysis (CVVHD) were performed. Finally, an analysis of the mechanism of action regarding classic and modern acid-base balance approaches is provided.

## Material and methods

### Ex vivo model

For all the experiments, an ex vivo model comprising 5 l fresh porcine blood connected to an extracorporeal support device (either ADVOS multi, CVVH, or CVVHD) was employed. For experiments with ADVOS multi or CVVHD, an additional continuous CO_2_ supply was added. Small modifications comprising the administration of different solutions were performed for each specific experiment (Fig. 1).

### Blood preparation

Fresh porcine blood was obtained from the local slaughterhouse (Münchner Schlachthof Betriebs GmbH, Munich, Germany) and prepared following a standard operation procedure (SOP) diluting it with modified Ringer’s solution (100 mmol/l NaCl, 3 mmol/l KCl, 1 mmol/l MgSO_4_.7H_2_O, 1.75 mmol/l CaCl_2_, 1200 mg/dl glucose) to a hematocrit of 36%, standard electrolyte concentrations, and normal blood gas values. 100,000 IU of heparin (Ratiopharm, Ulm, Germany) prevented coagulation. Blood was maintained at a constant temperature of 37 °C using a bath heater while stirring it at 130–180 rpm.

### ADVOS system

#### ADVOS multi

For the ADVOS system (ADVITOS GmbH, Munich, Germany), two SURELYZER PES-190 DH dialyzers (Nipro D.Med Germany GmbH, Hamburg, Germany) with blood and dialysate flowing co-currently were employed in the extracorporeal circuit. Blood flow can be adjusted between 100 and 400 ml/min. A dialysate flow of 800 ml/min was used throughout the study, which refers to the amount of fluid per minute being recirculated and detoxified in the ADVOS multi circuit by means of pH adjustments and filtration, instead of being discarded after a single pass. Variations of pH and composition are achieved using acidic and basic concentrates (see below). The concentrate flow (160 or 320 ml/min) determines the quantity of dialysate cleaned by convective transport in the ADVOS multi circuit. Toxins released from albumin or water-soluble toxins are separated from the albumin dialysate through two ELISIO-13H filters (Nipro D.Med Germany GmbH. Hamburg, Germany). Additionally, the concentrate flow refers to the amount of new fresh concentrate (i.e., mix of water and acidic and alkaline concentrates) pumped into the system.

#### CO_2_ administration for ADVOS multi and CVVHD experiments

For this ex vivo model, CO_2_ was continuously infused into the blood pool via an additional SURELYZER PES-190 DH dialyzer (Nipro D.Med Germany GmbH, Hamburg, Germany) connected to a CO_2_ gas supply (Linde AG, Munich, Germany). Contrary to a standard dialyzer setup, in the CO_2_ dialyzer, blood was circulated through the external side to reduce the pressure within the blood circuit. CO_2_ was supplied to the inner side of the CO_2_ dialyzer in a countercurrent flow via a pressure reducer (FMD 202, Linde AG, Munich, Germany) and a mass flow meter FMA-1618A (OMEGA Engineering, Deckenpfronn, Germany). The bottom outlet of the CO_2_ dialyzer was closed to avoid CO_2_ losses while the semipermeable membrane allowed the gas to diffuse freely into the blood.

#### Dialysate for ADVOS multi

In contrast to other hemodialysis methods, the ADVOS system does not use a fixed dialysate composition. Instead, two concentrates (acid and base) are automatically mixed in a specifically designed reservoir throughout the treatment depending on the desired dialysate pH (range 7.20–10.00). A higher dialysate pH setting means a higher basic/acidic concentrate ratio and thus higher sodium and lower chloride levels. Two 100 ml bottles of albumin 20% (Human Albumin 200 g/l, Baxter, Vienna, Austria) are added to the above dialysate mix via a specific port in the ADVOS multi device. The dialysate is furthermore supplemented with 40% glucose at an infusion rate of 70 ml/h to maintain glucose levels around 100 mg/dl in blood.

Acidic concentrate (AC) included H^+^, Na^+^, Cl^−^, HPO_4_^2−^, Mg^2+^, and Ca^2+^ while basic concentrate (BC) consisted primarily of OH^−^, Na^+^, and K^+^. Two versions of BC were employed. BC-Bic20 contained Na_2_CO_3_, while BC-Bic0 did not.

### Continuous veno-venous hemodialysis

#### NIKKISO DBB-03

The NIKKISO DBB-03 dialysis system (NIKKISO Europe GmbH, Langenhagen, Germany) consisted of a blood and dialysate circuit working as a single pass process without recirculating the dialysate. The dialysate flow (350 ml/min) determined the amount of dialysate being supplied and discarded. It was equipped with one SUREFLUX-25UX dialyzer (Nipro D.Med Germany GmbH, Hamburg, Germany) working with a countercurrent flow. Constant HCO_3_^−^ and Na^+^ concentrations in the dialysate were maintained by an integrated sodium bicarbonate cartridge (B. Braun Melsungen AG, Melsungen, Germany). Additionally, glucose (1 g/l), potassium (2 mmol/l), and calcium (1.5 mmol/l) were infused into the reservoir.

### Continuous veno-venous hemofiltration

#### Sartorius Haemoprocessor 40040

For standard CVVH system, the Haemoprocessor 40040 and its specific Plasma Line (Meise Medizintechnik GmbH, Schalksmühle, Germany) were connected to one SURELYZER PES-190 DH dialyzer (Nipro D.Med Germany GmbH, Hamburg, Germany). The multiPlus-CRRT solution containing 35 mmol/l of bicarbonate (Fresenius Medical Care, Bad Homburg, Germany) was employed as substitution solution in post-dilution mode. A blood flow of 200 ml/min was used for all experiments. The ultrafiltration and substitution flow rates were automatically adjusted by the device being always around 15 ml/min and 65 ml/min, respectively.

### Experimental design

Experiments were divided into three groups. First, blood was titrated with CO_2_ or lactic acid to achieve a blood pH range of 7.35–7.45 while being treated with ADVOS multi using different settings (set 1 and 2, respectively). Second, using a fixed CO_2_ or lactic acid supply, the performance of ADVOS multi vs. hemodialysis (CVVHD) or hemofiltration (CVVH) was compared (set 3 and set 4, respectively). Third, a hypercapnic acidosis was triggered in blood and further treated with ADVOS multi until recovery of normal blood gas values. Details for each of the experimental sets are summarized in Table 1.

**Table 1:** Experimental setting for each of the tests performed

	Set 1	Set 2	Set 3		Set 4		Set 5
Supply							
CO_2_ (ml/min)	(1)	(2)	110	110	(1)	No	27
Lactic acid (mmol/min)	No	(3)	No	No	0.5	0.5	No
Device settings							
Treatment	ADVOS	ADVOS	ADVOS	CVVHD	ADVOS	CVVH	ADVOS
Blood flow (ml/min)	100, 200, 400	100, 200, 400	400	400	200	200	200
Single pass dialysate flow (ml/min)	n.a.	n.a.	n.a.	350	n.a.	n.a.	n.a.
Recirculating dialysate flow (ml/min)*	800	800	800	n.a.	800	n.a.	800
Concentrate flow (ml/min)**	160, 320	320	320	n.a.	160	n.a.	160
Replacement solution flow (ml/min)	n.a.	n.a.	n.a.	n.a.	n.a.	65	n.a.
Ultrafiltration flow (ml/min)	n.a.	n.a.	n.a.	n.a.	n.a.	15	n.a.
Dialysate/replacement solution pH	7.5, 8.0, 8.5, 9.0, 10.0	7.5, 8.0, 8.5, 9.0	10.0	8.0	9.0	7.4	9.0
Dialyzer surface (m^2^)	2 × 1.9	2 × 1.9	2 × 1.9	2.5	2 × 1.9	1.9	2 × 1.9
Alkaline concentrate (during treatment phase)	BC-Bic20, BC-Bic0	BC-Bic20	BC-Bic0	n.a.	BC-Bic20	n.a.	BC-Bic0
Blood baseline levels (before treatment phase)							
pH	7.35–7.45	7.35–7.45	7.35–7.45	7.35–7.45	< 7.15	< 7.15	< 7.15
pCO_2_ (mmHg)	35–45	35–45	35–45	35–45	35–45	35–45	> 60
HCO_3_^−^ (mmol/l)	22–28	22–28	22–28	22–28	12–14	12–14	> 32
Lactate (mmol/l)	n.a.	n.a.	n.a.	n.a.	5–6	5–6	n.a.
Number of experiments performed***	3	3	6	6	3	3	2

*n.a.* not applicable

^(1)^CO_2_ supply was adjusted such that blood pH remained between 7.35 and 7.45

^(2)^CO_2_ was continuously infused on demand to maintain pCO_2_ levels between 35 and 45 mmHg

^(3)^A continuous 2% lactic acid solution was infused such that blood pH remained between 7.35 and 7.45

*The recirculating dialysate flow in the ADVOS system reflects the volume of dialysate that recirculates continuously (not discarded)

**The concentrate flow corresponds to the dialysate flow of a conventional single pass dialysis device and reflects the amount of dialysate used and discarded

***Experiments performed with each combination of blood flow, concentrate flow, and dialysate pH

#### Set 1: Influence of ADVOS multi operational settings on CO_2_ removal

In order to determine the influence of blood flow and dialysate composition on CO_2_ removal ability of the ADVOS system, different settings were tested during a continuous CO_2_ supply (Fig. 1). Briefly, 5 l of blood at physiological levels of pH (7.35–7.45), HCO_3_^−^ (22–28 mmol/l), and pCO_2_ (35–45 mmHg) was treated with ADVOS multi at experimental blood flows (Q_b_) of 100, 200, or 400 ml/min with co-currently recirculating dialysate at flows of 800 ml/min. At each Q_b_, dialysate pH was set to 7.5, 8.0, 8.5, and 9.0 using a concentrate flow (Q_c_) of 160 ml/min. At the highest Q_b_ of 400 ml/min, additional tests were carried out with a Q_c_ of 320 ml/min. All these experiments were carried out using the concentrates AC and BC-Bic20.

With the intention to test if different bicarbonate concentrations of the dialysate might affect CO_2_ removal, additional experiments with BC-Bic0 (without bicarbonate) instead of BC-Bic20 were carried out setting dialysate pH to 10.00.

Prior to blood hemodialysis, every dialyzer was primed with a 0.9% NaCl solution removing air before blood contact. The CO_2_ dialyzer was flushed with gas prior to and during NaCl and blood perfusion to create a positive pressure gradient which prevented liquids from entering the capillaries. Every test consisted of a 20-min stabilization period during which CO_2_ supply was adjusted such that blood pH remained between 7.35 and 7.45. This was followed by a 1-h treatment phase during which samples from the inlet (pre-dialyzer) and outlet (post-dialyzer) were analyzed by the blood gas analyzer GEM Premier 4000 (Instrumentation Laboratory, Munich, Germany) every 20 min. In addition, pH was measured by an InPro 3253 pH probe inserted into the blood container and M300 displayed it continuously (both Mettler Toledo, Greifersee, Switzerland).

To quantify CO_2_ removal in milliliters per minute, post-dialyzer TCO_2_ values in millimoles per liter were subtracted from pre-dialyzer values and this difference multiplied by the corresponding blood flow (Q_b_) and by the molar volume (V_m_) of CO_2_ at STP (22.4 ml/mmol) (Eq. 1). The fraction of TCO_2_ excreted as dissolved CO_2_ or HCO_3_^−^ was calculated likewise [19].

1 $$ {TCO}_2 removal\kern0.1em \left( ml/\mathit{\min}\right)=\left({TCO}_2 pre-{TCO}_2 post\right)\ast {Q}_b\ast {V}_m $$

#### Set 2: Influence of ADVOS multi operational settings on blood pH during continuous acid load

The same experimental design as for set 1 was performed to determine the influence of ADVOS parameters, with small modifications. Briefly, a continuous 2% lactic acid solution was used to titrate blood pH to 7.35–7.45. CO_2_, instead, was continuously infused on demand to maintain pCO_2_ levels between 35 and 45 mmHg. These experiments were only carried out with Q_c_ of 320 ml/min and with BC-Bic20.

For each experiment, the maximal combined acid supply resulting from lactic acid infusion and CO_2_ influx supply was calculated from the infusion rate of the pump (Volumat MC Agilia. Fresenius Kabi, Bad Homburg, Germany) and reading of the mass flow meter FMA-1618A (OMEGA Engineering, Deckenpfronn, Germany), respectively.

#### Set 3: ADVOS multi vs. CVVHD during continuous maximal CO_2_ supply

The maximum CO_2_ supply was determined previously in set 1 to be 110 ml/min (Additional file 1: Table S1). Blood was treated for 4 h with either the ADVOS multi or the CVVHD device NIKKISO DBB-03. The settings for each device are detailed in Table 1. Blood gas analysis was performed every 15 min.

#### Set 4: ADVOS multi vs. CVVH for the treatment of lactic acidosis in vitro

First, 5 l of fresh swine blood was subjected to CVVH with a substitution fluid with 10 mmol/l bicarbonate. Simultaneously, a 2% lactic acid solution was infused in order to reach a pH < 7.15, a pCO_2_ between 35 and 45 mmHg, HCO_3_^−^ levels between 12 and 14 mmol/l, and lactate of 5–6 mmol/l, which simulated a severe lactic acidosis. Blood was then treated with either ADVOS multi or CVVH for 1 h. Lactic acid infusion was maintained during the treatment phase. Blood was analyzed as described above.

#### Set 5: Treatment of hypercapnic acidosis in vitro with ADVOS multi

In this case, a hypercapnic acidosis was triggered first. Briefly, 27 ml/min CO_2_ was infused while a blood flow of 100 ml/min and a dialysate pH of 7.8 were set. During this phase, AC was combined with BC-Bic20. Once a pH < 7.15, a pCO_2_ > 60 mmHg, and HCO_3_^−^ levels >32 mmol/l were reached, both the settings and the BC were changed, and the treatment phase started. Blood was then treated with ADVOS multi until normal values of pH (7.35–7.45), pCO_2_ (35–45 mmHg), and HCO_3_^−^ (22–26 mmol/l) were detected. Blood values were analyzed as described above. CO_2_ was continuously supplied with the same flow of 27 ml/min.

### Acid-base balance according to Stewart

We obtained blood samples both at the inlet and the outlet of the dialyzers from experimental sets 1 and 2. The analysis of these data provides an understanding of pH variations attending to pCO_2_ and SID changes. In order to better understand this physicochemical method proposed by Stewart [20], several authors have tried to calculate specific values for the total concentration and the effective dissociation constant for plasma nonvolatile buffers. Constable suggested that “at normal pH (7.40), a 1-meq/l increase in SID will increase pH by 0.016, a 1-Torr increase in pCO_2_ will decrease pH by 0.009, and a 1 g/dl increase in total protein will decrease pH by 0.039” [21]. It is assumed that no variation on total protein content occurs in our setting as it cannot be lost in the dialyzer. Therefore, variations in [A_tot_] (i.e., total protein) are not considered within the equation that was employed to predict the resulting outlet pH.

Equation 2. Calculated pH in the outlet of the dialyzer based on measured values of pH, SID, and pCO_2_, adapted from [21]:

2 $$ \mathrm{pH}\ \mathrm{outlet}=\mathrm{pH}\ \mathrm{inlet}+0.016\times \left(\mathrm{SID}\ \mathrm{outlet}-\mathrm{SID}\ \mathrm{inlet}\right)-0.009\times \left({\mathrm{pCO}}_2\ \mathrm{outlet}-{\mathrm{pCO}}_2\ \mathrm{inlet}\right) $$

### Statistics

Experiments were performed at different settings between 3 and 6 times each covering a wide range of operational parameters. Student’s *t* test for paired samples was used to compare CO_2_ removal and acid supply between different ADVOS settings for experimental sets 1 and 2, respectively. A two-tailed *p* value lower than 0.05 was considered to indicate statistical significance. For correlations assessment, Pearson’s coefficient was employed. Data were documented and analyzed using Microsoft Excel and IBM SPSS 24.0 for Windows®, respectively. Data are presented as mean ± standard deviation (SD)

## Results

### Influence of ADVOS multi operational settings on CO_2_ removal

**Fig 2:**
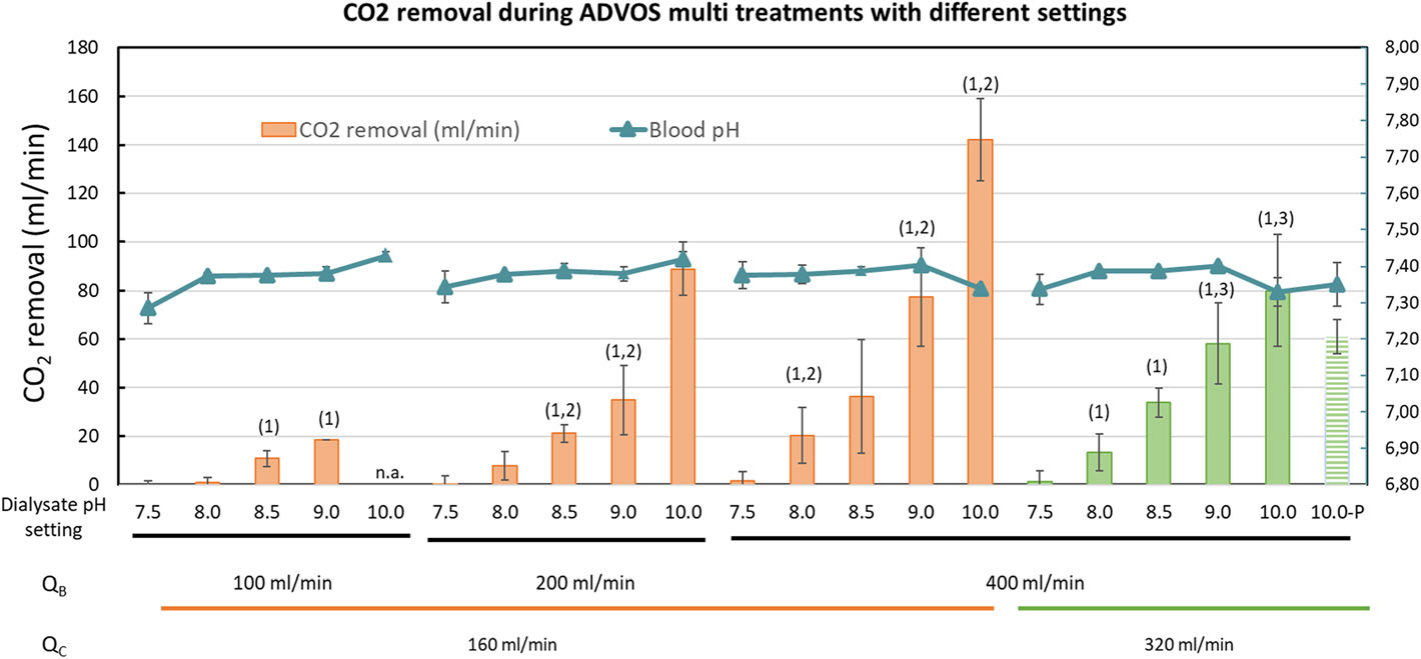
Results from blood gas analysis obtained after hemodialysis of swine blood under different treatment settings with ADVOS. Experiments with dialysate pH 10 were performed with the basic concentrate without Na_2_CO_3_ (BC-Bic 0). The value “10-P” corresponds to the experiments where CO_2_ was supplied as long as physiological blood gas values were maintained (pH 7.35–7.45, pCO_2_ 35–45 mmHg, HCO_3_^−^ 22–28 mmol/l) and not only blood pH. Mean ± SD.1, *p* < 0.05 for CO_2_ removal between consecutive dialysate pH settings among the same blood flow; 2, *p* < 0.05 for CO_2_ removal between consecutive blood flows among the same dialysate pH setting; 3, *p* < 0.05 for CO_2_ removal between consecutive concentrate flows among the same blood flow and dialysate pH setting

In this experimental design, where blood was titrated with CO_2_ to maintain a blood pH between 7.35 and 7.45, CO_2_ removal with ADVOS multi depended on three variables: (1) the amount of CO_2_ being supplied, (2) the blood flow, and (3) the dialysate composition (i.e., according to carbonate concentration and dialysate pH setting) (Fig. 2 and Additional file 1: Table S1). During experiments with BC-Bic20 and Q_c_ of 160 ml/min, higher Q_b_ resulted in higher CO_2_ elimination with an average of 77 ± 22 ml/min at Q_b_ 400 ml/min with a dialysate of pH 9.0. With the same dialysate pH, 35 and 19 ml/min of CO_2_ were removed at Q_b_ 200 and 100 ml/min, respectively. A lower dialysate pH setting (i.e., lower sodium and higher chloride) resulted in lower CO_2_ removal, independently of any other setting. In fact, the dialysate composition (i.e., the presence of albumin and sodium and chloride concentrations) together with the dialysate pH (but not dialysate pH alone) is responsible for blood pH correction.

At a Q_c_ of 160 ml/min compared to 320 ml/min, setting dialysate pH to 9.0 resulted in significantly higher blood HCO_3_^−^ (57.0 ± 10.2 vs. 38.5 ± 2.8 mmol/l) and pCO_2_ (103 ± 17 vs. 74 ± 4 mmHg), which is correlated with the higher amount of CO_2_ removed (Additional file 1: Table S1).

Increasing the Q_c_ from 160 ml/min to 320 ml/min means doubling the convective transport, which results in faster removal of substances diffused from blood (e.g., bicarbonate). Therefore, at higher concentrate flows, a more efficient concentration gradient between blood and dialysate is available, resulting, in this case, in lower blood baseline levels of HCO_3_^−^ with a Q_c_ of 320 ml/min in comparison to a Q_c_ of 160 ml/min. Lower blood HCO_3_^−^ can buffer less acid in blood (i.e., less CO_2_ can be supplied without altering blood pH), which, due to the lower total CO_2_ concentration in blood (i.e., lower pCO_2_ at the inlet of the dialyzer), it was translated in a lower CO_2_ removal in our experimental setting. Nevertheless, this is an artefactual result caused by the experimental design as not Q_c_, but Q_b_, CO_2_ supply, and dialysate pH setting affect the CO_2_ removal capacity.

In line with these results, using a carbonate-free dialysate BC-Bic0 with dialysate pH set to 10, a maximum removal of 142 ml/min CO_2_ was achieved during ADVOS treatment with Q_b_ of 400 ml/min and Q_c_ of 160 ml/min. However, with this setting, pCO_2_ and HCO_3_^−^ were extremely above physiological levels (117 ± 5 mmHg and 62.8 ± 3.4 mmol/l, respectively). Thus, blood was then titrated with CO_2_ only as long as every blood gas value was maintained within a physiological range (including pCO_2_ and HCO_3_^−^). This setting allowed a CO_2_ removal of 61 ml/min using a Q_b_ of 400 ml/min and a Q_c_ of 320 ml/min. This was only possible with a basic concentrate without carbonate. In fact, similar CO_2_ removal rates were achieved with the same flows and with dialysate pH 9 with the BC-Bic20 concentrate (58 ml/min), but blood gas levels were above physiological values in this case (Fig. 2 and Additional file 1: Table S1).

Variations between the inlet (pre) and the outlet (post) of the dialyzers for SID, pCO_2_, HCO_3_^−^, and pH are shown in Additional file 1: Table S1.

### Influence of ADVOS multi operational settings on blood pH during continuous acid load

**Fig. 3:**
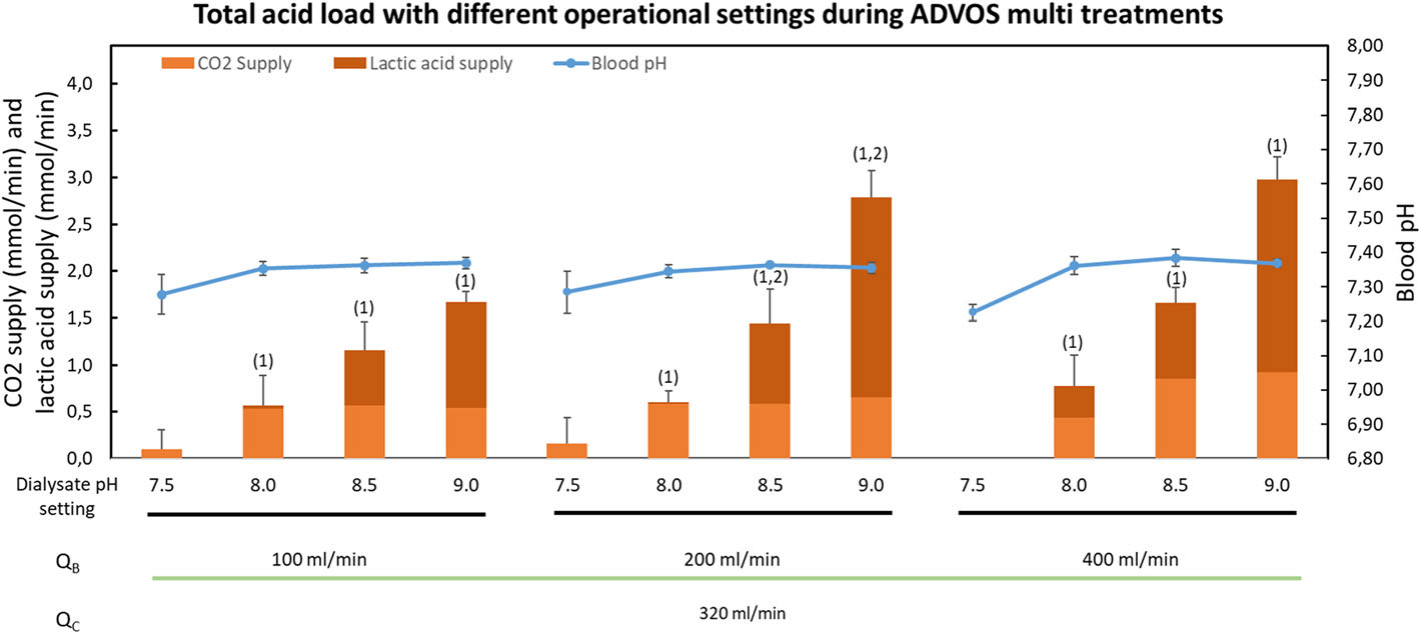
Total acid load (CO_2_ + lactic acid) with different operational settings during ADVOS multi treatments. CO_2_ and lactic acid were supplied to maintain pCO_2_ and blood pH between 35–45 mmHg and 7.35–7.45, respectively. A supply of 1 mmol/l of CO_2_ corresponds to 22.5 ml/min in normal conditions. 1, *p* < 0.05 for CO_2_ removal between consecutive dialysate pH settings among the same blood flow; 2, *p* < 0.05 for CO_2_ removal between consecutive blood flows among the same dialysate pH setting.

Experiments with ADVOS multi employing different operational settings showed that higher blood flows and dialysate pH settings were able to allow higher acid loads. The influence of each parameter is shown in Fig. 3. These results show a low level of CO_2_ removal needed (< 1 mmol/min or 22.5 ml/min) to maintain pCO_2_ stable (35–45 mmHg) in those cases where CO_2_ elimination is not required.

Variations between the inlet (pre) and the outlet (post) of the dialyzers for SID, pCO_2_, HCO_3_^−^, and pH are shown in Additional file 1: Table S2.

### ADVOS multi vs. CVVHD during continuous maximal CO_2_ supply

**Fig. 4:**
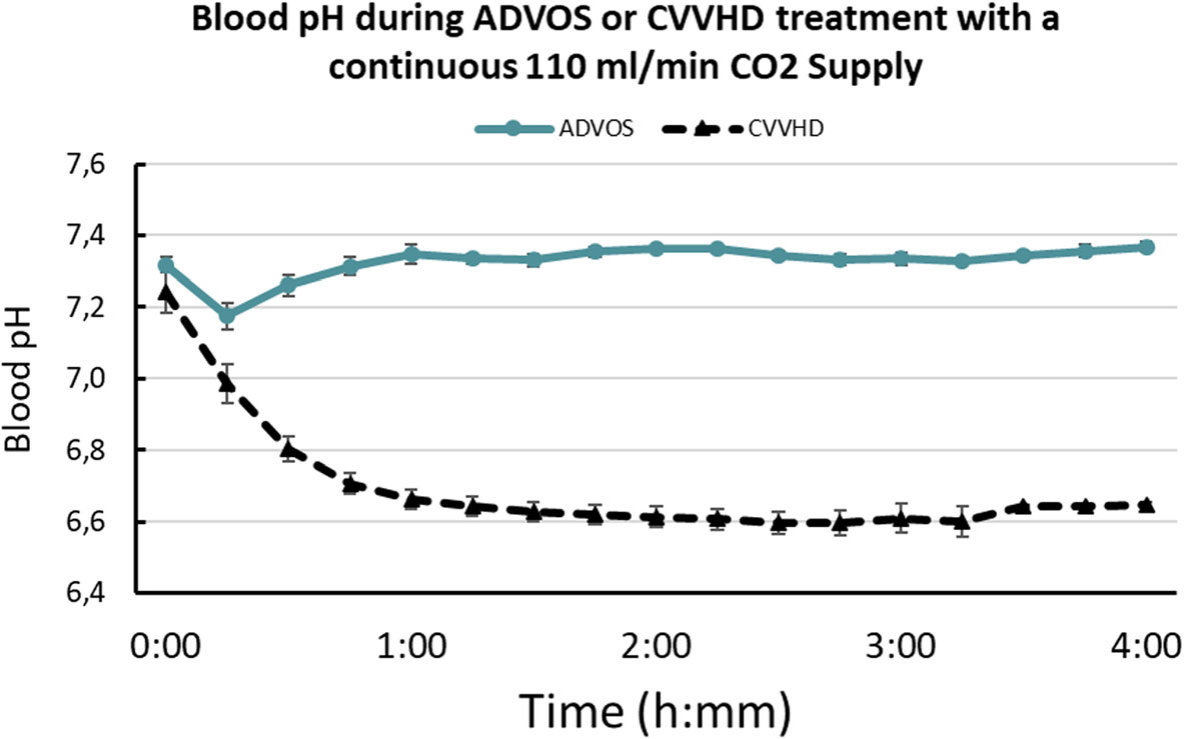
Comparison of blood pH between a conventional hemodialysis (NIKKISO DBB-03) and the ADVOS system 4-h treatment under 110 ml/min CO_2_ supply. Error bars represent SD. *n* = 6

Using a carbonate-free dialysate with a pH of 10, ADVOS multi removed 79 ± 1 ml/min TCO_2_ on average during 4 h of continuous supply of 110 ml/min CO_2_. Blood pCO_2_ (66 ± 9 mmHg) and HCO_3_^−^ (33.1 ± 0.1 mmol/l) levels were stable throughout the experiments. The ADVOS device was able to maintain pH stable and within the physiological range (Fig. 4) while post-dialyzer pH remained always below 8.

In contrast, despite identical CO_2_ influx, blood pH dropped already 15 min after circulating blood through the NIKKISO renal dialysis device reaching a constant pH of around 6.60 after 1 h. Post-dialyzer pH remained below 7 during the experiment.

### Treatment of metabolic acidosis in vitro: ADVOS multi vs. CVVH

**Fig. 5:**
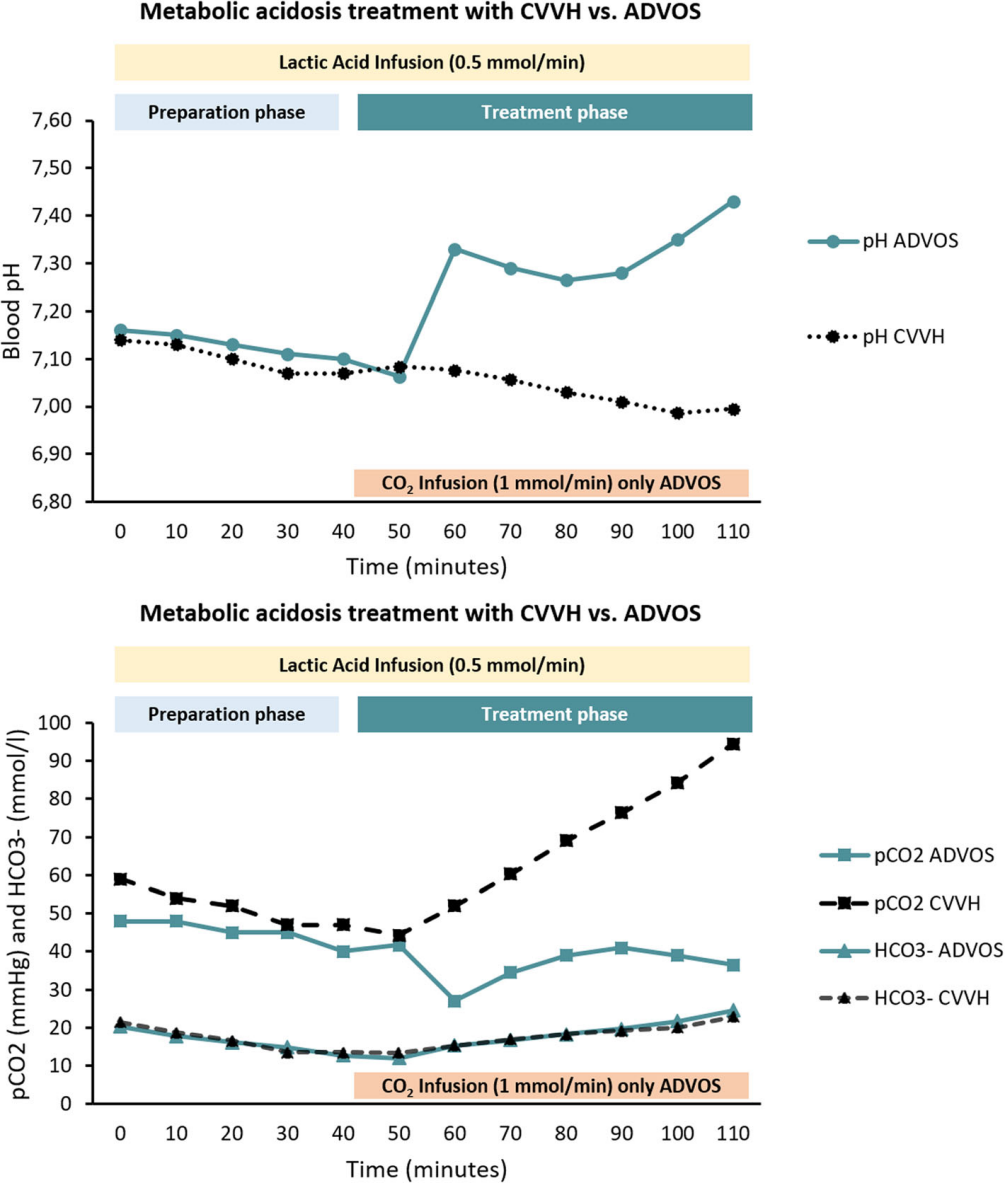
Comparison of the course of blood pH (up), HCO_3_^−^, and pCO_2_ (bottom) during treatments with ADVOS and CVVH. A metabolic acidosis was triggered in blood reaching baseline values before treatment of pH < 7.2, HCO_3_^−^ < 14 mmol/l, and pCO_2_ of 45 mmHg (preparation phase). Then, for 1 h, either a conventional hemofiltration using a commercially available substitution fluid with 35 mmol/l bicarbonate or a treatment with ADVOS multi with a dialysate pH of 9.00 was performed. Lactic acid was continuously supplied to maintain lactate levels over 5 mmol/l

Once metabolic acidosis was triggered (minute 40, Fig. 5), bicarbonate therapy during CVVH was able to normalize HCO_3_^−^ levels. However, as expected, due to the lack of ventilation, pCO_2_ was correspondingly elevated (> 90 mmHg) resulting in an even lower pH (< 7.00) after the treatment. Conversely, ADVOS multi normalized pH and bicarbonate levels in less than 1 h. Due to the high CO_2_ removal ability of the ADVOS system when dialysate pH is set to 9.0, even 15 ml/min of CO_2_ was additionally provided during the treatment with ADVOS multi to maintain blood pH between 7.35 and 7.45.

### Treatment of hypercapnic acidosis in vitro with ADVOS multi

**Fig. 6:**
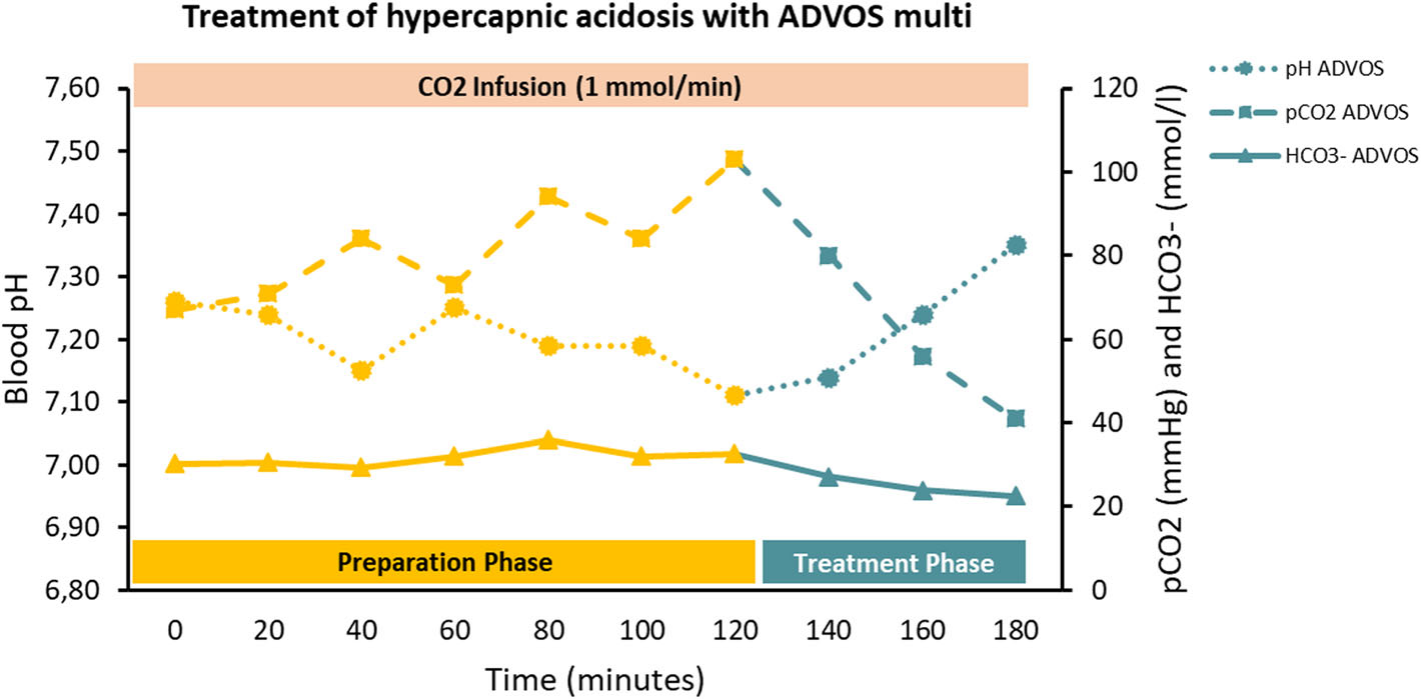
Course of pH, pCO_2_, and HCO_3_^−^ in blood during ADVOS multi treatment with a continuous supply of 27 ml/min of CO_2_. During the preparation phase (yellow), a respiratory acidosis was triggered while this was corrected during the treatment phase (green)

Using a basic concentrate without Na_2_CO_3_ (BC-Bic 0), ADVOS multi was able to restore a hypercapnic acidosis in vitro in less than 30 min using a Q_b_ of 200 ml/min, a Q_c_ of 160 ml/min, and dialysate pH set to 9.0 during a continuous supply of 27 ml/min of CO_2_. After changing the settings (dialysate pH 7.8 vs. 9.0) and the basic concentrate (BC-Bic 20 vs. BC-Bic 0), values of pH (7.12 vs. 7.35), pCO_2_ (99 vs. 40 mmHg), and HCO_3_^−^ (32.7 vs. 22.6 mmol/l) returned to physiological standards (Fig. 6).

### Acid-base balance according to Stewart

As shown in Additional file 1: Tables S1 and S2, the higher the pCO_2_ reduction, the higher the pH increase that can be achieved in blood, describing a direct correlation for more than 200 blood samples in both experimental sets 1 and 2 (Additional file 2: Figure S1 *r*^2^ = 0.812 and Additional file 2:Figure S2 *r*^2^ = 0.935, respectively).

To note, to increase the dialysate pH, the ratio “basic concentrate/acidic concentrate” increases. Attending to the composition of these solutions, an increase in the basic/acidic concentrate ratio results in a dialysate with higher sodium and lower chloride concentrations. Indeed, an increase in SID was observed for set 1 (Additional file 1: Table S1), but not for set 2 due to the lactate addition (Additional file 1: Table S2).

Finally, we were able to predict pH variations solely by measuring SID and pCO_2_ changes (Additional file 2: Figures S1 and S2; Additional file 3: Figures S3 and S4; Additional file 4: Figures S5 and S6). The calculated values correlated perfectly with measured values (*r*^2^ = 0.98). Variations in total protein are not expected since no albumin loss occurs in the dialyzer.

## Discussion

In the present study, the ability of an albumin hemodialysis system (ADVOS multi) to correct hypercapnic and lactic acidosis in vitro has been demonstrated. Different settings can be varied in this device (blood flow, concentrate flow, carbonate content, and dialysate pH), allowing different rates of CO_2_ removal or acid load. This is only possible due to the presence of albumin in the dialysate, which permits to alter the composition of the dialysate (including the strong ion difference and the CO_2_ content). A concentrate gradient between blood and dialysate for electrolytes or bicarbonate is then possible, allowing the correction of acidosis from hypercapnic or metabolic origin.

### Lung support through CO_2_ removal

The lung removes CO_2_ directly, thanks to the fast transformation of H^+^ + HCO_3_^−^ through carbonic anhydrase into CO_2_ gas and water. However, in blood, CO_2_ is mainly found as HCO_3_^−^. ADVOS reduces pCO_2_ through the removal of HCO_3_^−^ by forming a concentration gradient between blood and dialysate. Additionally, the high dialysate pH helps to reduce the H^+^ concentration in blood and thus increase the pH. This is possible due to the presence of albumin in the dialysate. Albumin increases the buffer capacity via a protonation of its imidazole side chain [22], which contains several buffering residues of histidine [23, 24]. Preliminary in-house studies indicate that a dialysate of pH 9 containing two 100 ml bottles of albumin 20% increased the buffer capacity by 35% compared to the same dialysate without albumin (Additional file 5: Figure S7).

Bearing this in mind, first, a higher blood flow may account for a higher HCO_3_^−^ concentration gradient between blood and dialysate (i.e., 400 ml/min). Second, a higher dialysate pH (with a higher SID) allows a higher decrease in H^+^ concentration (i.e., dialysate pH 10). Third, lower (or none) dialysate carbonate levels permit a more effective convective transport in the ADVOS multi circuit (Fig. 1). Finally, this convective transport will be faster insofar a higher concentrate flow is set (i.e., 320 ml/min) (Fig. 2 and Additional file 1: Table S1). Even at lower blood and concentrate flows (200 and 160 ml/min, respectively) and with dialysate pH set to 9, the use of a dialysate without carbonate allowed a correction of hypercapnic acidosis in vitro in less than 1 h (Fig. 6).

### Kidney support through HCO_3_^−^ generation

The renal compensatory mechanism during a respiratory acidosis tries to increase the acid excretion into urine and the HCO_3_^−^ resorption into blood [25]. When using a bicarbonate containing dialysate with high pH, this mechanism is mimicked by ADVOS.

This can be explained following the CO_2_ equilibrium in Eq. 3. Carbonic acid, or CO_2_ in its gas form, is converted to HCO_3_^−^ and H^+^. The reduction of H^+^ concentration in blood forces the equilibrium to the right of the equation, increasing HCO_3_^−^ even as it is removed from blood, and consequently, CO_2_ is transferred down its concentration gradient from the intracellular space (i.e., correcting intracellular acidosis) into the blood and into the dialysate in the form of HCO_3_^−^. This means that in the absence of adequate ventilation, as simulated in our ex vivo model through elevated CO_2_ supply without additional oxygenation, ADVOS multi could “imitate” the renal compensatory mechanism for acidemia control [26].

3 $$ {\mathrm{H}}_2{\mathrm{CO}}_3\leftrightarrow {\mathrm{CO}}_2+{\mathrm{H}}_2\mathrm{O}\leftrightarrow {{\mathrm{H}\mathrm{CO}}_3}^{-}+{\mathrm{H}}^{+} $$

In the case of ADVOS, the higher the dialysate pH setting, the higher the H^+^ concentration reduction is achieved (Fig. 2). This results into HCO_3_^−^ generation, which helps to correct HCO_3_^−^ levels during metabolic acidosis (Fig. 5). This is only possible if a concomitant pCO_2_ reduction is achieved, which does not occur during conventional renal replacement therapy or bicarbonate infusion. In fact, in the absence of adequate ventilation (i.e., CO_2_ removal), a metabolic acidosis can turn into a hypercapnic acidosis (Fig. 5), which cannot be corrected with conventional CVVHD (Fig. 4).

### The Stewart model to explain acid-base balance with the ADVOS system

We analyzed if the observed changes could also be explained by the mathematical model proposed by Stewart [20] and its revision by others [27–29], who showed that three independent variables are responsible for determining the pH in plasma: PaCO_2_, plasma weak acids (i.e., phosphate and albumin), and the SID as the difference between fully dissociated plasma anions and cations. Therefore, neither the H^+^ movement nor the buffering effect of HCO_3_^−^ is necessary or sufficient to explain acid-base regulation. In any case, our data can be also explained using this model, as shown in Additional files.

Taking into account the conclusions obtained by Constable [21], blood pH could be predicted solely by changes in total protein, pCO_2_, and SID. Applying this to our data demonstrated a perfect correlation between the measured and the calculated pH in the outlet of the dialyzer (Additional file 2: Figures S1 and S2; Additional file 3: Figures S3 and S4; Additional file 4: Figures S5 and S6). Indeed, SID variations were correlated with dialysate pH variations, specifically at high values of 10.0. To reach such a dialysate pH, a higher rate of basic/acidic concentrate is needed, which provides higher Na^+^ and lower Cl^−^, and can therefore result into SID reductions in blood through the dialyzer. Moreover, the presence of albumin as a weak acid facilitates this process.

Although SID and pCO_2_ are considered independent variables by Stewart, it has been suggested that an interdependency between both values might exist [30]. Langer et al. observed that the greater the variation in pCO_2_, the greater the reduction in plasma SID. We might reach the same conclusion when, as this group did, quartiles of pCO_2_ variations are analyzed and plotted against the corresponding SID variations (*r*^2^ = 0.991; Additional file 6: Figure S8). However, if raw data are drawn, no correlation is observed (*r*^2^ = 0.190; Additional file 7: Figure S9).

### Rationale for multi organ support with ADVOS during acidosis

Using either the classical or the modern approach, this work should serve as a proof of concept of the ability of the ADVOS therapy to correct acid-base disturbances. As described above, the lungs (i.e., CO_2_) and kidneys (i.e., NH_4_^+^, HCO_3_^−^, for the classic approach or Na^+^, Cl^−^ for the Stewart model) are usually defined to be responsible for acid-base control. However, the liver plays also an important role (i.e., metabolism of organic acid anions like citrate and certain amino acids) [31, 32] and can also be supported by ADVOS. Indeed, acidemia and metabolic acidosis are associated with poor outcome in cirrhosis patients, as demonstrated by Drolz and colleagues in a cohort of 178 critically ill patients with liver cirrhosis and acute on chronic liver failure [33].Therefore, attention should not only be paid to a specific organ. In addition, the majority of the cases of acidosis reflect a mixed nature, involving both a metabolic and a respiratory component [2]. In view of this, a multiple organ approach seems to be needed while facing acidosis, where the variety of adjustable parameters of the ADVOS multi might play an important role.

### Limitations and justification of the ex vivo model

Although our results are encouraging, our work is limited by its in vitro nature, the selection of parameters, and the number of experiments performed. Nevertheless, so as to serve as a proof of concept, this experimental setting is adequate based on the following: (1) the different parameters analyzed and varied (i.e., pCO_2_, HCO_3_^−^, lactate) to resemble different types of acidosis, (2) the possibility to control the concrete amount of acid load (i.e., CO_2_ and/or lactic acid) being supplied, and (3) the analysis of inlet and outlet measurements to describe the course of blood values along the dialyzer. These encouraging results need now to be confirmed in the clinical setting.

## Conclusions

In conclusion, the ADVOS albumin hemodialysis system was able to remove 61 ml/min while maintaining blood gas values in the physiological range or up to 142 ml/min CO_2_ in hypercapnic conditions at low blood flow without the need of a gas phase. Blood pH was maintained stable within the physiological range of 7.35–7.45 by the albumin-containing dialysate. Moreover, during continuous lactic acid addition, up to 3 mmol/min of acid load was compensated. The major determinants to stabilize blood pH were blood flow, dialysate composition, and blood bicarbonate levels. The mechanism of action of ADVOS multi can be explained using either the classical acid-base balance model or the newer Stewart approach. This feature in combination with the previously demonstrated ability to eliminate water-soluble and protein-bound toxins may be of valuable help in the management of critically ill patients with multiple organ failure.

## Supplementary information

**Additional file 1: Table S1.** Results from blood gas analysis obtained after hemodialysis of swine blood under different treatment settings with ADVOS and different CO2 supply during experimental Set 1. Samples were taken in the inlet and outlet of the dialyzer at the same time. Mean ± S.D. **Table S2.** Results from blood gas analysis obtained after hemodialysis of swine blood under different treatment settings with ADVOS and different lactic acid supply during experimental Set 2. Samples were taken in the inlet and outlet of the dialyzer at the same time. Mean ± S.D.

**Additional file 2: Figure S1 and S2.** Correlation between variations of inlet and outlet pCO_2_ with measured or calculated variations in blood pH during experimental Set 1 and Set 2, respectively. The black line shows values obtained from BGA. The green line shows values calculated according to the Equation 2 (see methods 2.7). According to these data, the removal of CO_2_ and the corresponding decrease on pCO_2_ accounts for the elevation of blood pH. The higher the CO_2_ removal, the higher the pH increase in blood that can be achieved.

**Additional file 3: Figure S3 and S4.** Representation of all the BGA tests performed during the experiments from Set 1 and Set 2, respectively. The black line shows values obtained from blood. The green line shows calculated values considering variations (inlet – outlet) from pCO_2_ and SID according Equation 2 (see methods 2.7). It is assumed that no variation on total protein content occurs as it cannot be lost in the dialyzer. Therefore, variations in [A_tot_] are not considered within the equation. These results show, that taking into account variations in pCO2 and SID along the dialyzer, the resulting pH at the outlet can be predicted following the calculations suggested by Stewart [29].

**Additional file 4: Figure S5 and S6.** Correlation of the measured and calculated pH variations between the inlet and the outlet of the dialyzer (ΔpH = pH_outlet_ – pH_inlet_) during experimental Set 1 and 2, respectively. Measured values were obtained from BGA while calculated values were obtained according to the Equation 2. Each line accounts for a combination of different ADVOS settings (blood flow/concentrate flow). As demonstrated for Supplementary Figure 3 and 4, for each of the settings, there is a correlation between measured and calculated values according to the Stewart approach.

**Additional file 5: Figure S7.** Buffer capacity of a dialysate containing 20 mmol/l sodium bicarbonate with or without albumin (2 g/dl). The buffer capacity (β) is defined as the moles of an acid or base necessary to change the pH of a solution by 1, divided by the pH change and the volume of buffer in liters.

**Additional file 6: Figure S8.** Analysis of SID variations (outlet – inlet) according to quartiles of pCO_2_ variation (outlet – inlet). As shown in [30]. Mean ± S.D.

**Additional file 7: Figure S9.** Correlation between SID variations (outlet – inlet) and pCO_2_ variation (outlet – inlet) using raw data. These data show, that in our experiments there is no interdependence between SID and pCO_2_ variation, contrary to what is described in [30]. Using quartiles for pCO_2_ variation as shown in Supp. Figure 8, an artefactual correlation might be created.

## Data Availability

The datasets used and/or analyzed during the current study are available from the corresponding author on reasonable request.

## Abbreviations

ADVOS: ADVanced Organ Support
AKI: Acute kidney injury
ARDS: Acute respiratory distress syndrome
CO_2_: Carbon dioxide
COPD: Chronic obstructive pulmonary disease
HCO_3_: Bicarbonate
ICU: Intensive care unit
MOF: Multiple organ failure
MOST: Multiple organ support therapy
Na_2_CO_3_: Sodium carbonate
NaHCO_3_: Sodium bicarbonate
PaCO_2_: Arterial partial pressure of carbon dioxide
pCO_2_: Partial pressure of carbon dioxide
Q_b_: Blood flow
Q_c_: Concentrate flow
SD: Standard deviation
SID: Strong ion difference
SOFA: Sepsis-related organ failure assessment
SOP: Standard operating procedure
TCO_2_: Total content of CO_2_

## References

[1] Tiruvoipati R, Pilcher D, Buscher H, Botha J, Bailey M (2017). Effects of hypercapnia and hypercapnic acidosis on hospital mortality in mechanically ventilated patients. Crit Care Med..

[2] Jung B, Rimmele T, Le Goff C, Chanques G, Corne P, Jonquet O (2011). Severe metabolic or mixed acidemia on intensive care unit admission: incidence, prognosis and administration of buffer therapy. A prospective, multiple-center study. Crit Care..

[3] Kim HJ, Son YK, An WS (2013). Effect of sodium bicarbonate administration on mortality in patients with lactic acidosis: a retrospective analysis. PLoS One..

[4] Kluge S, de HG, Jarczak D, Nierhaus A, Fuhrmann V (2018). Lactic acidosis - update 2018. Dtsch Med Wochenschr..

[5] Critical care nephrology: Springer; 1998.

[6] Kellum JA, Song M, Li J (2004). Science review: Extracellular acidosis and the immune response: clinical and physiologic implications. Crit Care..

[7] Zampieri FG, Kellum JA, Park M, Ranzani OT, Barbeiro HV, de SHP (2014). Relationship between acid-base status and inflammation in the critically ill. Crit Care..

[8] Lardner A (2001). The effects of extracellular pH on immune function. J Leukoc Biol..

[9] Mitchell JH, Wildenthal K, Johnson RL (1972). JR. The effects of acid-base disturbances on cardiovascular and pulmonary function. Kidney Int..

[10] Stringer W, Wasserman K, Casaburi R, Porszasz J, Maehara K, French W (1994). Lactic acidosis as a facilitator of oxyhemoglobin dissociation during exercise. J Appl Physiol (1985)..

[11] Lejeune P, Brimioulle S, Leeman M, Hallemans R, Melot C, Naeije R (1990). Enhancement of hypoxic pulmonary vasoconstriction by metabolic acidosis in dogs. Anesthesiology..

[12] Farber MO, Szwed JJ, Dowell AR, Strawbridge RA (1976). The acute effects of respiratory and metabolic acidosis on renal function in the dog. Clin Sci Mol Med..

[13] Tournadre JP, Allaouchiche B, Malbert CH, Chassard D (2000). Metabolic acidosis and respiratory acidosis impair gastro-pyloric motility in anesthetized pigs. Anesth Analg..

[14] Engstrom M, Schott U, Romner B, Reinstrup P (2006). Acidosis impairs the coagulation: a thromboelastographic study. J Trauma..

[15] Al-Chalabi A, Matevossian E, V Thaden A-K, Luppa P, Neiss A, Schuster T (2013). Evaluation of the Hepa Wash(R) treatment in pigs with acute liver failure. BMC Gastroenterol..

[16] Al-Chalabi A, Matevossian E, von Thaden A, Schreiber C, Radermacher P, Huber W (2017). Evaluation of an ADVanced Organ Support (ADVOS) system in a two-hit porcine model of liver failure plus endotoxemia. Intensive Care Med Exp..

[17] Huber W, Henschel B, Schmid R, Al-Chalabi A (2017). First clinical experience in 14 patients treated with ADVOS: a study on feasibility, safety and efficacy of a new type of albumin dialysis. BMC Gastroenterol..

[18] Fuhrmann VH, Jarczak D, Boenisch O, Kluge S (2018). ADVOS reduces liver and kidney disease markers and corrects acidosis: the Hamburg experience. Critical Care..

[19] May AG, Sen A, Cove ME, Kellum JA, Federspiel WJ (2017). Extracorporeal CO2 removal by hemodialysis: in vitro model and feasibility. Intensive Care Med Exp..

[20] Stewart PA (1983). Modern quantitative acid-base chemistry. Can J Physiol Pharmacol..

[21] Constable PD (2001). Total weak acid concentration and effective dissociation constant of nonvolatile buffers in human plasma. J Appl Physiol (1985)..

[22] Kaneko K, Chuang VTG, Minomo A, Yamasaki K, Bhagavan NV, Maruyama T, Otagiri M (2011). Histidine146 of human serum albumin plays a prominent role at the interface of subdomains IA and IIA in allosteric ligand binding. IUBMB Life..

[23] Abe H (2000). Role of histidine-related compounds as intracellular proton buffering constituents in vertebrate muscle. Biochemistry (Mosc)..

[24] Szebedinszky C, Gilmour KM (2002). The buffering power of plasma in brown bullhead (Ameiurus nebulosus). Comp Biochem Physiol B Biochem Mol Biol..

[25] Skelton LA, Boron WF, Zhou Y. Acid-base transport by the renal proximal tubule. J Nephrol. 2010;23 Suppl 16:S4-18. PMC4699187 21170887

[26] Dorman PJ, Sullivan WJ, Pitts RF (1954). The renal response to acute respiratory acidosis. J Clin Invest..

[27] Figge J, Mydosh T, Fencl V (1992). Serum proteins and acid-base equilibria: a follow-up. J Lab Clin Med..

[28] Figge J, Rossing TH, Fencl V (1991). The role of serum proteins in acid-base equilibria. J Lab Clin Med..

[29] Kellum JA, Paul WG, WG EP (2009). Stewart’s textbook of acid-base.

[30] Langer T, Scotti E, Carlesso E, Protti A, Zani L, Chierichetti M (2015). Electrolyte shifts across the artificial lung in patients on extracorporeal membrane oxygenation: Interdependence between partial pressure of carbon dioxide and strong ion difference. J Crit Care..

[31] Scheiner B, Lindner G, Reiberger T, Schneeweiss B, Trauner M, Zauner C, Funk G-C (2017). Acid-base disorders in liver disease. J Hepatol..

[32] Gullo A, Häussinger D (1998). The role of the liver in acid-base regulation: anaesthesia, pain, intensive care and emergency medicine - A.

[33] Drolz A, Horvatits T, Roedl K, Rutter K, Brunner R, Zauner C (2018). Acid-base status and its clinical implications in critically ill patients with cirrhosis, acute-on-chronic liver failure and without liver disease. Ann Intensive Care..

