# Supplementary material for: Respiratory and metabolic acidosis correction with the ADVanced Organ Support system
Source: Intensive Care Med Exp. 2019 Sep 18;7:56. doi: 10.1186/s40635-019-0269-7 (PMC6751235; doi:10.1186/s40635-019-0269-7)
Supplement: Supplementary file 1 — Additional file 1: Table S1. Results from blood gas analysis obtained after hemodialysis of swine blood under different treatment settings with ADVOS and different CO2 supply during experimental Set 1. Samples were taken in the inlet and outlet of the dialyzer at the same time. Mean ± S.D. Table S2. Results from blood gas analysis obtained after hemodialysis of swine blood under different treatment settings with ADVOS and different lactic acid supply during experimental Set 2. Samples were taken in the inlet and outlet of the dialyzer at the same time. Mean ± S.D. [file 40635_2019_269_MOESM1_ESM.docx]

**Table S1.** Results from blood gas analysis obtained after hemodialysis of swine blood under different treatment settings with ADVOS and different CO_2_ supply during experimental Set 1. Samples were taken in the inlet and outlet of the dialyzer at the same time. Mean ± S.D.

| **Q_b_ (ml/min)** | **Q_c_ (ml/min)** | **Dialysate pH setting** | **CO_2_ supply (ml/min)** | **Blood pH** | | | **pCO_2_ (mmHg)** | | | **HCO_3_^-^ (mmol/l)** | | **SID (mEq/l)** | | **CO_2_ removal (ml/min)** |
| --- | --- | --- | --- | --- | --- | --- | --- | --- | --- | --- | --- | --- | --- | --- |
|  |  |  |  | **inlet** | **outlet** | **inlet** | | **outlet** | **inlet** | | **outlet** | **inlet** | **outlet** |  |
| 100 | 160 | 7.50 | 1 ± 1 | 7.29 ± 0.04 | 7.33 ± 0.05 | 50 ± 5 | | 49 ± 5 | 19.9 ± 0.5 | | 20.3 ± 0.5 | 32 ± 1 | 30 ± 1 | 0 ± 1 |
|  |  | 8.00 | 10 ± 0 | 7.37 ± 0.01 | 7.67 ± 0.13 | 47 ± 3 | | 29 ± 7 | 23.7 ± 1.7 | | 23.8 ± 1.3 | 32 ± 1 | 32 ± 1 | 1 ± 2 |
|  |  | 8.50 | 20 ± 1 | 7.38 ± 0.01 | 7.86 ± 0.04 | 62 ± 3 | | 22 ± 2 | 32.1 ± 1.7 | | 28.6 ± 1.1 | 36 ± 1 | 35 ± 1 | 11 ± 3 |
|  |  | 9.00 | 34 ± 2 | 7.38 ± 0.02 | >8.00 | 80 ± 5 | | 12 ± 2 | 43.8 ± 3.5 | | 34.5 ± 1.2 | 41 ± 2 | 36 ± 2 | 19 ± 2 |
|  |  | 10.00^§^ | 72 ± 1 | 7.43 ± 0.01 | >8.00 | 66 ± 10 | | n.a. | 43.7 ± 6.5 | | n.a. | 43 ± 3 | 27 ± 2 | n.a. |
| 200 | 160 | 7.50 | 1 ± 0 | 7.34 ± 0.04 | 7.36 ± 0.03 | 47 ± 3 | | 48 ± 3 | 20.8 ± 1.2 | | 20.7 ± 0.8 | 30 ± 2 | 31 ± 1 | 0 ± 3 |
|  |  | 8.00 | 15 ± 1 | 7.38 ± 0.01 | 7.54 ± 0.03 | 53 ± 5 | | 39 ± 3 | 25.6 ± 2.3 | | 24.8 ± 1.2 | 32 ± 2 | 32 ± 3 | 8 ± 6 |
|  |  | 8.50 | 28 ± 0 | 7.39 ± 0.02 | 7.70 ± 0.05 | 65 ± 3 | | 33 ± 2 | 33.3 ± 2.1 | | 29.6 ± 1.5 | 36 ± 2 | 35 ± 2 | 21 ± 35 |
|  |  | 9.00 | 58 ± 5 | 7.38 ± 0.02 | 7.95 ± 0.05 | 87 ± 9 | | 21 ± 4 | 45.4 ± 4.6 | | 39.6 ± 3.6 | 41 ± 2 | 38 ± 2 | 35 ± 14 |
|  |  | 10.00^§^ | 90 ± 3 | 7.42 ± 0.02 | 7.98 ± 0.02 | 97 ± 4 | | 12 ± 5 | 53.7 ± 2.0 | | 38.3 ± 5.2 | 50 ± 1 | 39 ± 2 | 89 ± 11 |
| 400 | 160 | 7.50 | 4 ± 2 | 7.38 ± 0.04 | 7.36 ± 0.03 | 45 ± 6 | | 48 ± 4 | 21.4 ± 1.3 | | 21.2 ± 1.2 | 32 ± 1 | 33 ± 1 | 1 ± 4 |
|  |  | 8.00 | 23 ± 3 | 7.38 ± 0.03 | 7.44 ± 0.03 | 57 ± 6 | | 48 ± 4 | 28.7 ± 3.2 | | 26.8 ± 2.3 | 34 ± 1 | 34 ± 1 | 20 ± 11 |
|  |  | 8.50 | 46 ± 8 | 7.39 ± 0.01 | 7.57 ± 0.02 | 74 ± 5 | | 48 ± 3 | 39.7 ± 5.9 | | 36.6 ± 2.3 | 39 ± 1 | 38 ± 1 | 36 ± 23 |
|  |  | 9.00 | 90 ± 23 | 7.40 ± 0.01 | 7.83 ± 0.10 | 103 ± 17 | | 39 ± 5 | 57.0 ± 10.2 | | 50.3 ± 3.8 | 48 ± 4 | 46 ± 6 | 77 ± 20 |
|  |  | 10.00^§^ | 114 ± 4 | 7.34 ± 0.01 | 7.84 ± 0.04 | 117 ± 5 | | 29 ± 2 | 62.8 ± 3.4 | | 49.6 ± 4.1 | 54 ± 2 | 47 ± 1 | 142 ± 17 |
|  | 320 | 7.50 | 1 ± 1 | 7.34 ± 0.04 | 7.33 ± 0.03 | 50 ± 5 | | 49 ± 5 | 20.5 ± 0.5 | | 23.7 ± 0.3 | 29 ± 1 | 30 ± 1 | 1 ± 5 |
|  |  | 8.00 | 19 ± 2 | 7.39 ± 0.00 | 7.47 ± 0.02 | 53 ± 2 | | 42 ± 1 | 25.3 ± 25.3 | | 24.2 ± 1.0 | 32 ± 1 | 31 ± 1 | 13 ± 8 |
|  |  | 8.50 | 39 ± 7 | 7.39 ± 0.01 | 7.56 ± 0.03 | 63 ± 3 | | 41 ± 1 | 30.8 ± 30.8 | | 27.7 ± 0.8 | 34 ± 3 | 34 ± 1 | 34 ± 6 |
|  |  | 9.00 | 63 ± 4 | 7.40 ± 0.01 | 7.74 ± 0.02 | 74 ± 4 | | 33 ± 2 | 38.5 ± 2.8 | | 33.2 ± 2.6 | 37 ± 2 | 36 ± 3 | 58 ± 17 |
|  |  | 10.00^§^ | 110 ± 3 | 7.33 ± 0.04 | 7.77 ± 0.10 | 66 ± 9 | | 19 ± 7 | 33.1 ± 0.1 | | 23.9 ± 3.4 | 38 ± 1 | 39 ± 2 | 80 ± 23 |
|  |  | 10.00^§ ⱡ^ | 69+ ± 2 | 7.35 ± 0.06 | 7.69 ± 0.01 | 36 ± 3 | | 12 ± 1 | 20.2 ± 1.0 | | 13.7 ± 1.2 | 34 ± 1 | 29 ± 1 | 61 ± 7 |

**§** Basic concentrate without Na_2_CO_3_ was employed (BC-Bic 0).

**ⱡ** CO_2_ supply as long as physiological blood gas values are maintained (pH 7.35-7.45; pCO_2_ 35-45 mmHg; HCO_3_^-^ 22-28 mmol/l).

**Q_b_**: blood flow; **Q_c_**: concentrate flow; **SID**: Strong Ion Difference. SID = Na^+^ + K^+^ + 2*Ca^2+^ - Cl^-^ - Lactate. All in mEq/l.

**n.a.**: not applicable. CO_2_ removal was not calculated since post-dialyzer blood gas values were out of range for BGA.

**Table S2.** Results from blood gas analysis obtained after hemodialysis of swine blood under different treatment settings with ADVOS and different lactic acid supply during experimental Set 2. Samples were taken in the inlet and outlet of the dialyzer at the same time. Mean ± S.D.

| **Q_b_ (ml/min)** | **Q_c_ (ml/min)** | **Dialysate pH setting** | **CO_2_ supply (ml/min)** | **Lactic acid supply (mmol/min)** | **Blood pH** | | **pCO_2_ (mmHg)** | | **HCO_3_^-^ (mmol/l)** | | **SID (mEq/l)** | | **CO_2_ removal (ml/min)** |
| --- | --- | --- | --- | --- | --- | --- | --- | --- | --- | --- | --- | --- | --- |
|  |  |  |  |  | **inlet** | **outlet** | **inlet** | **outlet** | **inlet** | **outlet** | **inlet** | **outlet** |  |
| 100 | 320 | 7.50 | 2 ± 5 | 0.00 ± 0.00 | 7.28 ± 0.06 | 7.20 ± 0.07 | 44 ± 5 | 44 ± 6 | 20.4 ± 0.8 | 20.8 ± 0.4 | 30 ± 1 | 30 ± 1 | < 1 |
|  |  | 8.00 | 12 ± 6 | 0.03 ± 0.04 | 7.35 ± 0.02 | 7.52 ± 0.09 | 44 ± 4 | 30 ± 6 | 24.5 ± 1.6 | 24.0 ± 0.9 | 32 ± 1 | 32 ± 1 | 2 ± 4 |
|  |  | 8.50 | 13 ± 4 | 0.58 ± 0.19 | 7.36 ± 0.02 | 7.84 ± 0.05 | 47 ± 3 | 16 ± 2 | 26.7 ± 0.8 | 27.4 ± 1.0 | 33 ± 1 | 34 ± 1 | 1 ± 2 |
|  |  | 9.00 | 12 ± 2 | 1.12 ± 0.18 | 7.37 ± 0.02 | >8.00 | 47 ± 3 | 8 ± 1 | 27.5 ± 1.2 | n.a. | 32 ± 1 | 34 ± 2 | n.a. |
| 200 | 320 | 7.50 | 4 ± 6 | 0.00 ± 0.00 | 7.28 ± 0.06 | 7.23 ± 0.11 | 45 ± 6 | 50 ± 12 | 20.9 ± 0.8 | 20.5 ± 1.0 | 30 ± 1 | 30 ± 1 | 1 ± 5 |
|  |  | 8.00 | 13 ± 2 | 0.02 ± 0.04 | 7.35 ± 0.02 | 7.49 ± 0.04 | 48 ± 2 | 33 ± 3 | 25.9 ± 1.0 | 25.4 ± 1.1 | 33 ± 1 | 33 ± 1 | 5 ± 4 |
|  |  | 8.50 | 13 ± 1 | 0.86 ± 0.40 | 7.36 ± 0.01 | 7.65 ± 0.06 | 47 ± 1 | 25 ± 3 | 26.9 ± 0.8 | 27.2 ± 0.7 | 33 ± 1 | 34 ± 1 | 2 ± 4 |
|  |  | 9.00 | 15 ± 0 | 2.13 ± 0.27 | 7.36 ± 0.02 | 7.90 ± 0.07 | 47 ± 1 | 15 ± 2 | 26.2 ± 1.1 | 29.8 ± 0.9 | 32 ± 2 | 35 ± 1 | < 1 |
| 400 | 320 | 7.50 | 0 ± 0 | 0.00 ± 0.00 | 7.23 ± 0.02 | 7.21 ± 0.02 | 48 ± 2 | 51 ± 2 | 20.3 ± 0.2 | 20.3 ± 0.8 | 30 ± 1 | 30 ± 1 | < 1 |
|  |  | 8.00 | 10 ± 2 | 0.33 ± 0.19 | 7.36 ± 0.03 | 7.45 ± 0.01* | 42 ± 2 | 35 ± 2 | 24.1 ± 1.0 | 24.4 ± 0.7 | 32 ± 2 | 32 ± 2 | < 1 |
|  |  | 8.50 | 19 ± 2 | 0.80 ± 0.06 | 7.38 ± 0.03 | 7.55 ± 0.02* | 46 ± 2 | 31 ± 2 | 27.9 ± 0.8 | 27.2 ± 0.6 | 33 ± 2 | 34 ± 2 | 10 ± 10 |
|  |  | 9.00 | 21 ± 1 | 2.05 ± 0.19 | 7.37 ± 0.01 | 7.72 ± 0.03* | 49 ± 1 | 22 ± 1 | 27.9 ±0.8 | 29.4 ± 0.4 | 34 ± 2 | 35 ± 2 | < 1 |

CO_2_ supply as long as physiological blood gas values are maintained (pH 7.35-7.45; pCO_2_ 35-45 mmHg; HCO_3_^-^ 22-28 mmol/l).

**Q_b_**: blood flow; **Q_c_**: concentrate flow; **SID**: Strong Ion Difference. SID = Na^+^ + K^+^ + 2*Ca^2+^ - Cl^-^ - Lactate. All in mEq/l.

**n.a.**: not applicable. CO_2_ removal was not calculated since post-dialyzer blood gas values were out of range for BGA.
