# Supplementary figures and images for: Respiratory and metabolic acidosis correction with the ADVanced Organ Support system
Source: Intensive Care Med Exp. 2019 Sep 18;7:56. doi: 10.1186/s40635-019-0269-7 (PMC6751235; doi:10.1186/s40635-019-0269-7)

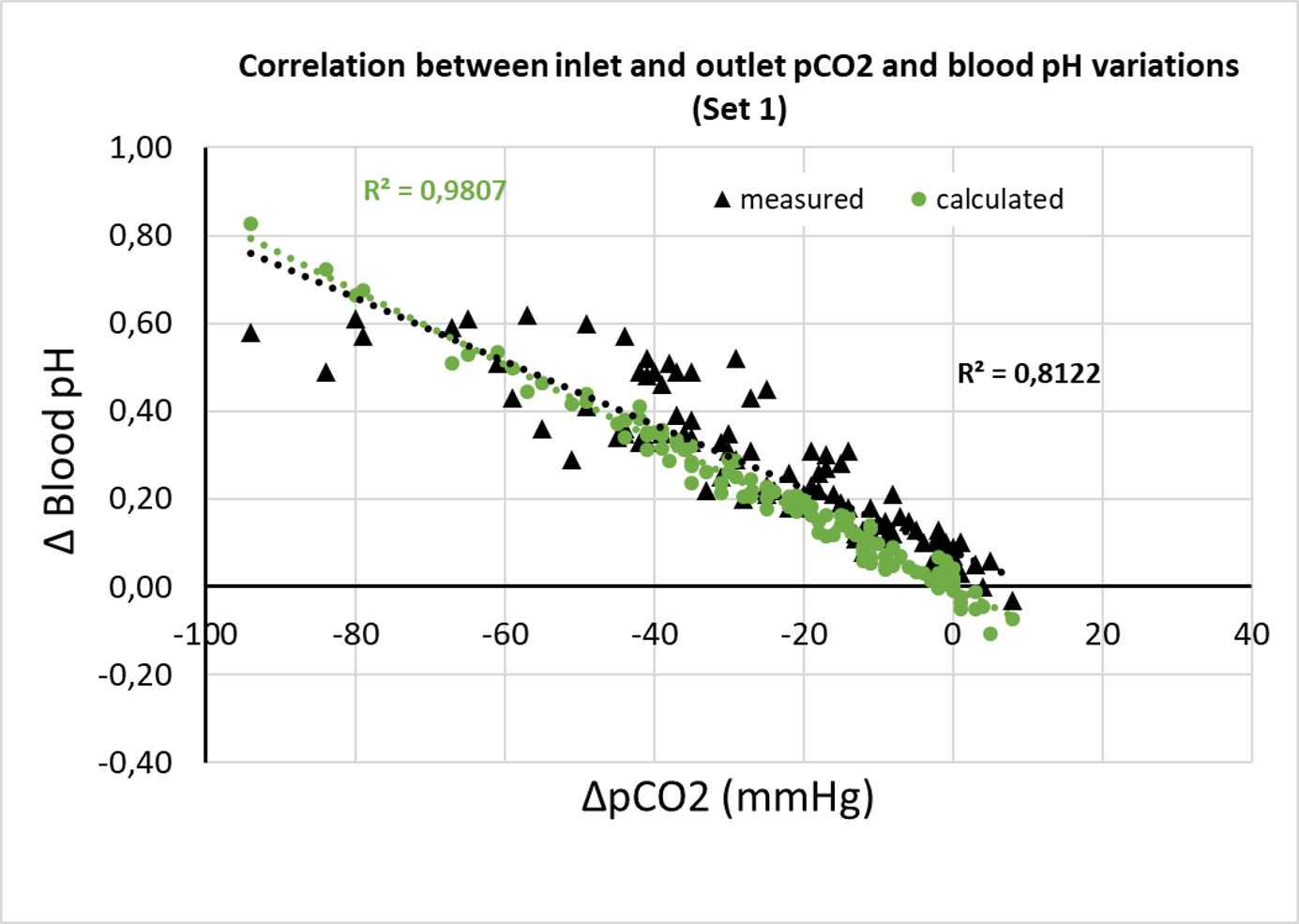

Supplement: Supplementary file 2 — Additional file 2: Figure S1 and S2. Correlation between variations of inlet and outlet pCO2 with measured or calculated variations in blood pH during experimental Set 1 and Set 2, respectively. The black line shows values obtained from BGA. The green line shows values calculated according to the Equation 2 (see methods 2.7). According to these data, the removal of CO2 and the corresponding decrease on pCO2 accounts for the elevation of blood pH. The higher the CO2 removal, the higher the pH increase in blood that can be achieved. [file 40635_2019_269_MOESM2_ESM.zip › Figure S1.tif]

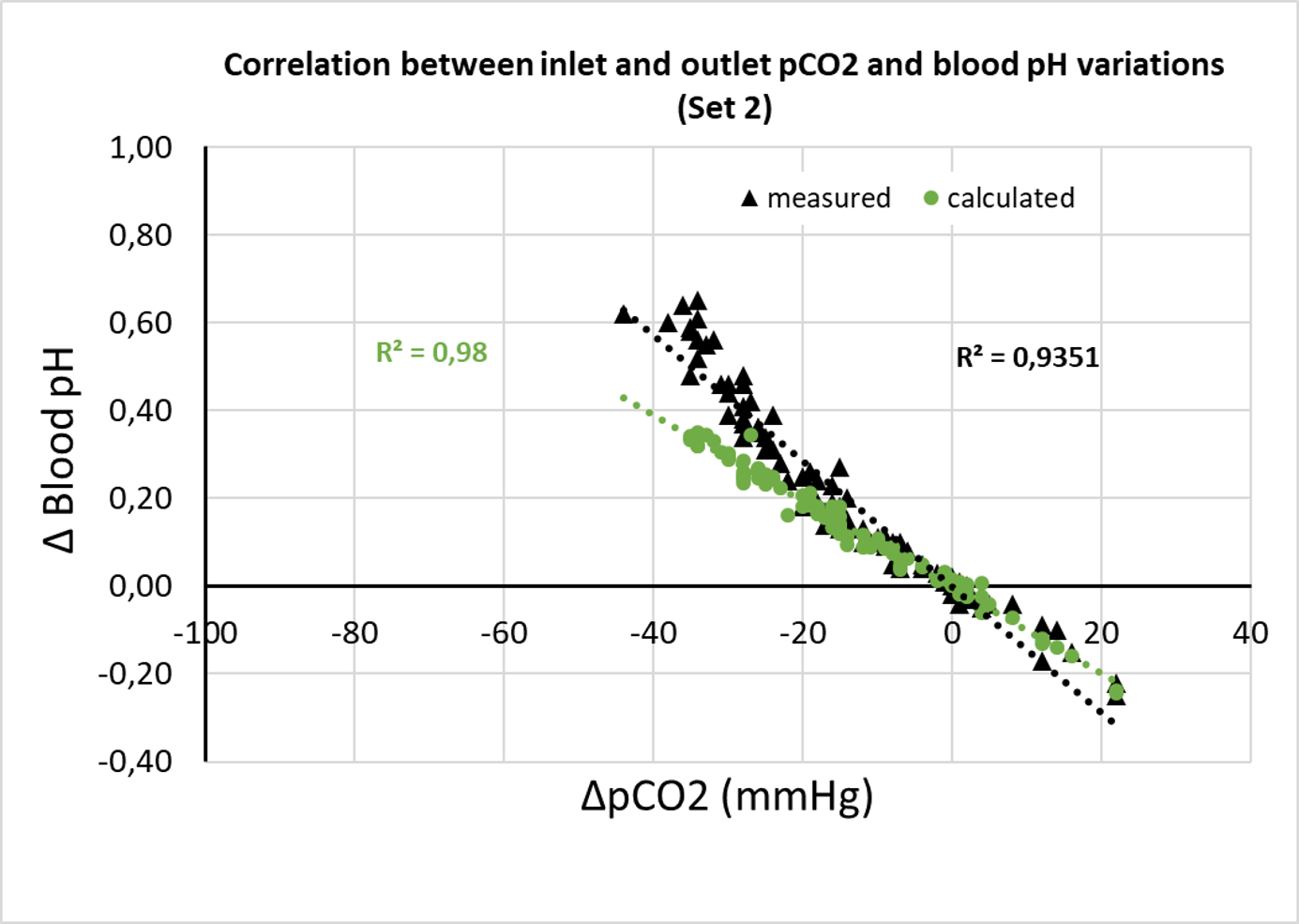

Supplement: Supplementary file 2 — Additional file 2: Figure S1 and S2. Correlation between variations of inlet and outlet pCO2 with measured or calculated variations in blood pH during experimental Set 1 and Set 2, respectively. The black line shows values obtained from BGA. The green line shows values calculated according to the Equation 2 (see methods 2.7). According to these data, the removal of CO2 and the corresponding decrease on pCO2 accounts for the elevation of blood pH. The higher the CO2 removal, the higher the pH increase in blood that can be achieved. [file 40635_2019_269_MOESM2_ESM.zip › Figure S2.tif]

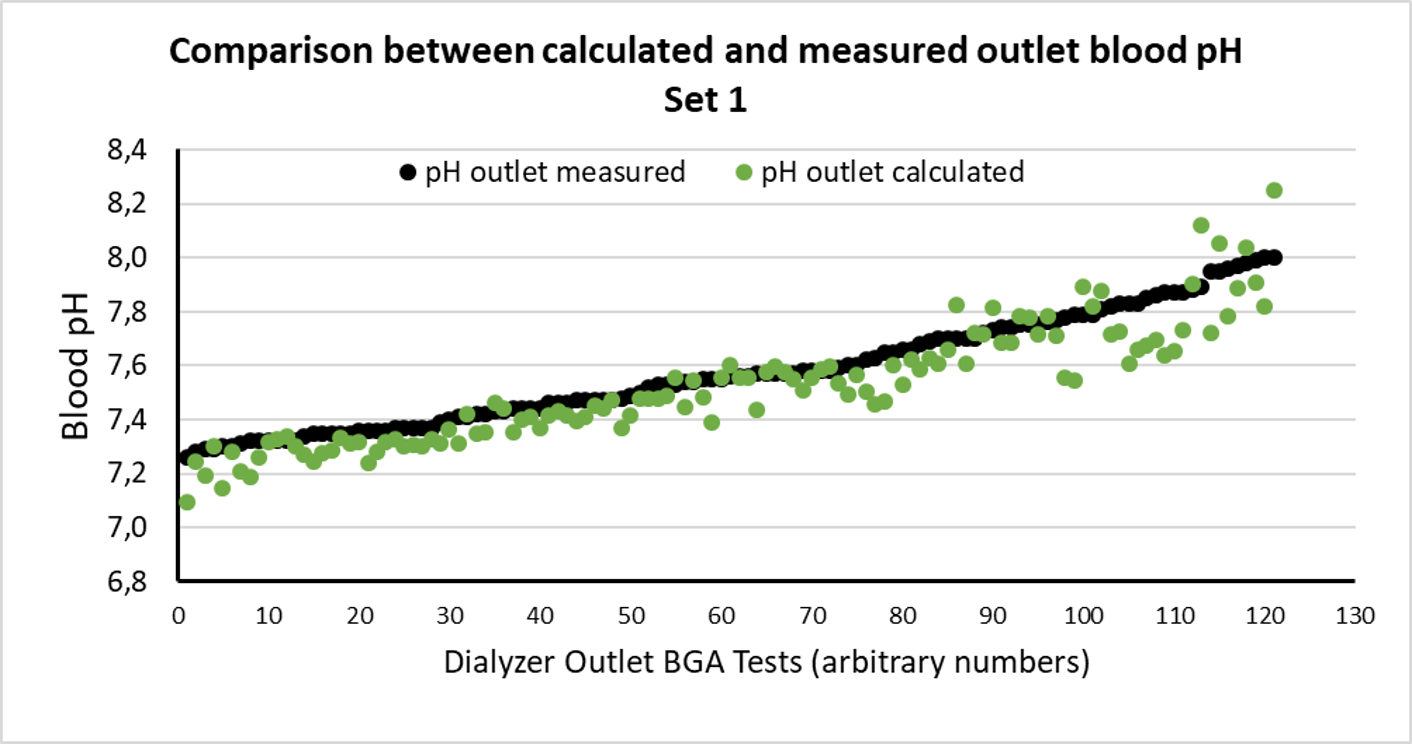

Supplement: Supplementary file 3 — Additional file 3: Figure S3 and S4. Representation of all the BGA tests performed during the experiments from Set 1 and Set 2, respectively. The black line shows values obtained from blood. The green line shows calculated values considering variations (inlet – outlet) from pCO2 and SID according Equation 2 (see methods 2.7). It is assumed that no variation on total protein content occurs as it cannot be lost in the dialyzer. Therefore, variations in [Atot] are not considered within the equation. These results show, that taking into account variations in pCO2 and SID along the dialyzer, the resulting pH at the outlet can be predicted following the calculations suggested by Stewart [29]. [file 40635_2019_269_MOESM3_ESM.zip › Figure S3.tif]

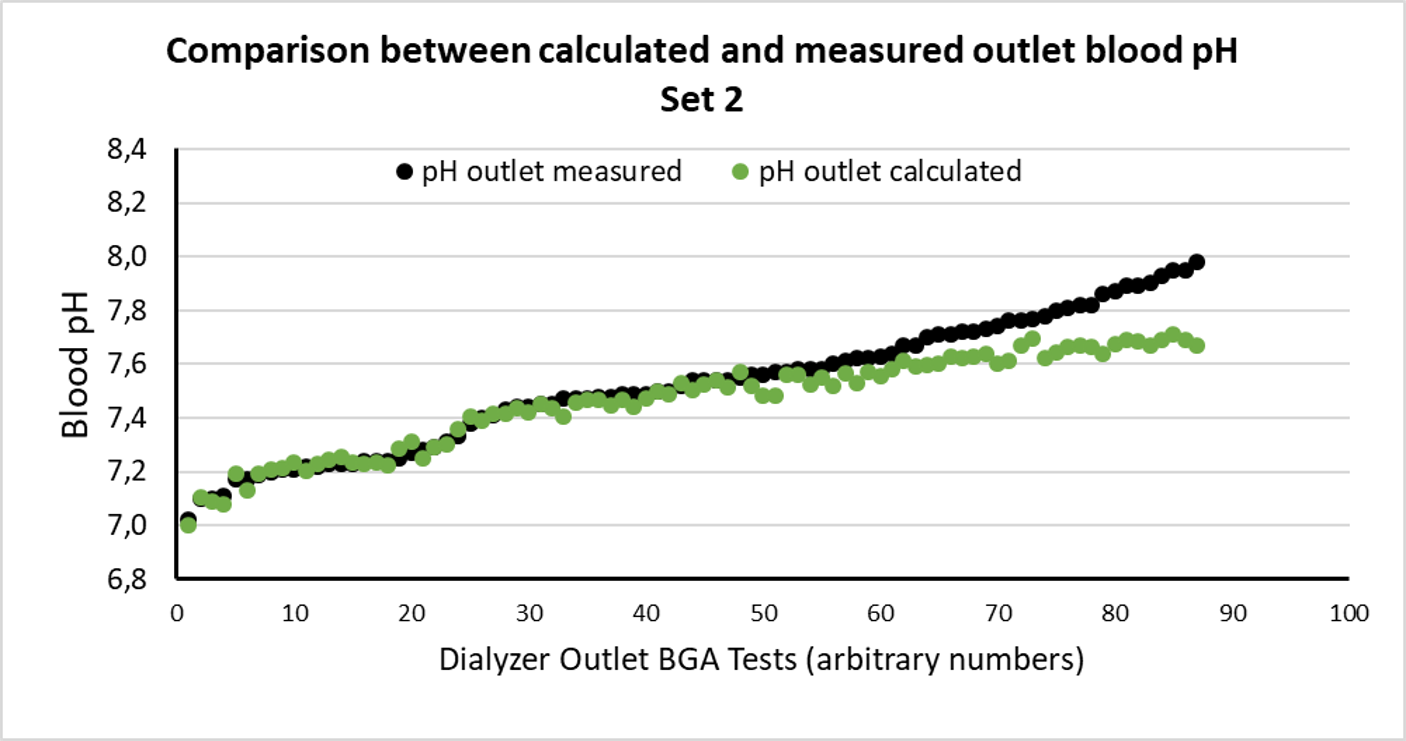

Supplement: Supplementary file 3 — Additional file 3: Figure S3 and S4. Representation of all the BGA tests performed during the experiments from Set 1 and Set 2, respectively. The black line shows values obtained from blood. The green line shows calculated values considering variations (inlet – outlet) from pCO2 and SID according Equation 2 (see methods 2.7). It is assumed that no variation on total protein content occurs as it cannot be lost in the dialyzer. Therefore, variations in [Atot] are not considered within the equation. These results show, that taking into account variations in pCO2 and SID along the dialyzer, the resulting pH at the outlet can be predicted following the calculations suggested by Stewart [29]. [file 40635_2019_269_MOESM3_ESM.zip › Figure S4.tif]

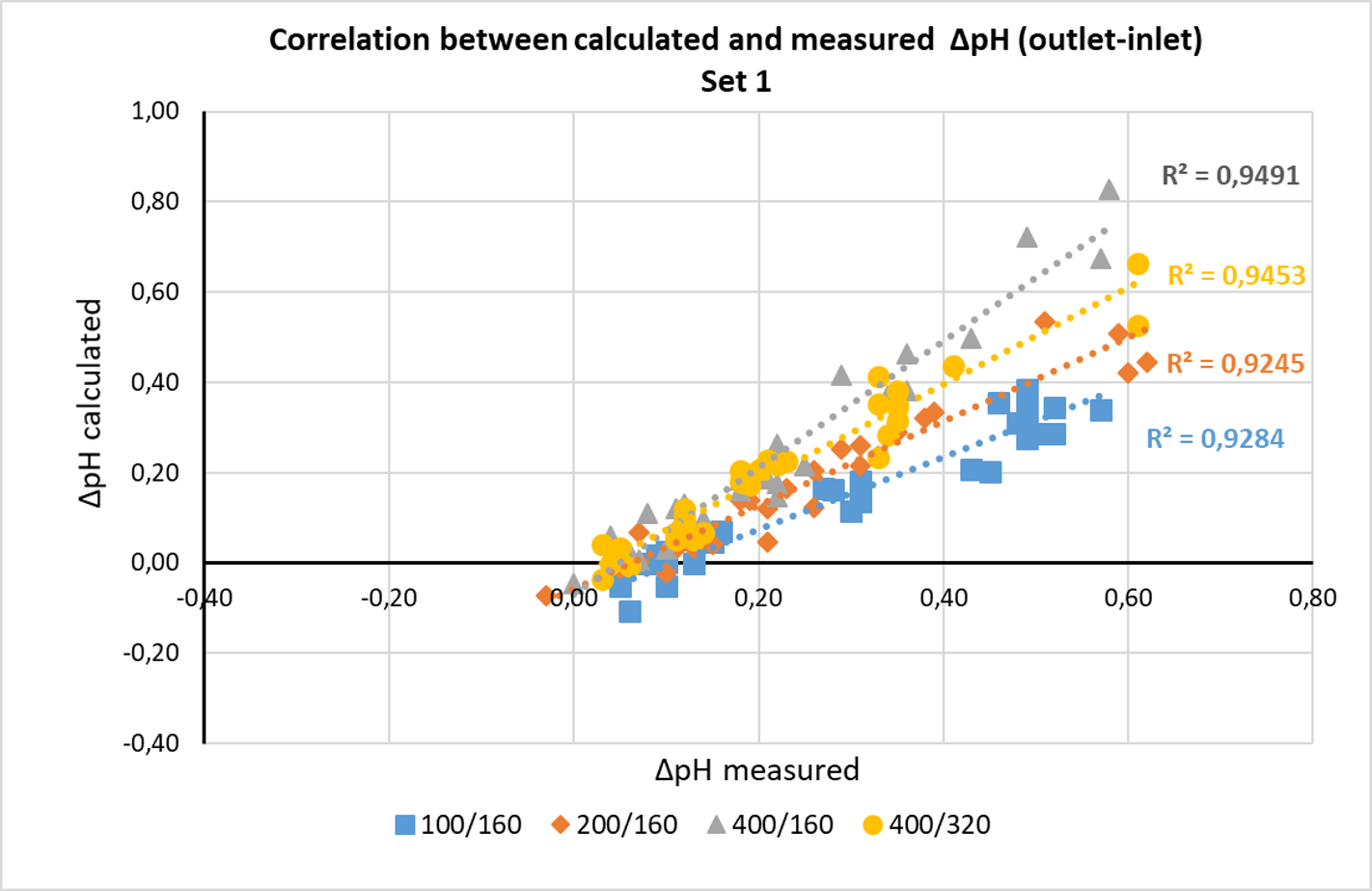

Supplement: Supplementary file 4 — Additional file 4: Figure S5 and S6. Correlation of the measured and calculated pH variations between the inlet and the outlet of the dialyzer (ΔpH = pHoutlet – pHinlet) during experimental Set 1 and 2, respectively. Measured values were obtained from BGA while calculated values were obtained according to the Equation 2. Each line accounts for a combination of different ADVOS settings (blood flow/concentrate flow). As demonstrated for Supplementary Figure 3 and 4, for each of the settings, there is a correlation between measured and calculated values according to the Stewart approach. [file 40635_2019_269_MOESM4_ESM.zip › Figure S5.tif]

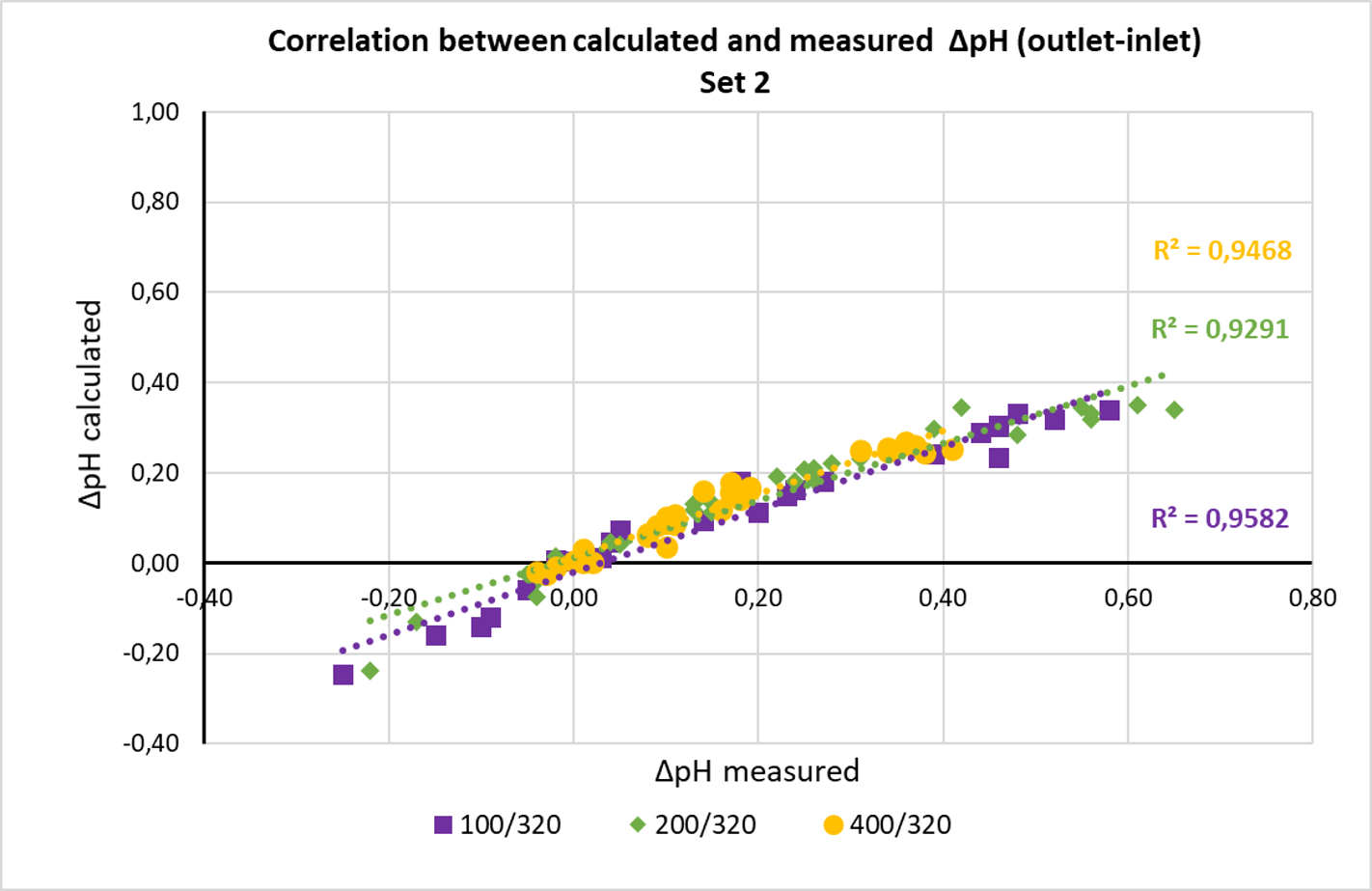

Supplement: Supplementary file 4 — Additional file 4: Figure S5 and S6. Correlation of the measured and calculated pH variations between the inlet and the outlet of the dialyzer (ΔpH = pHoutlet – pHinlet) during experimental Set 1 and 2, respectively. Measured values were obtained from BGA while calculated values were obtained according to the Equation 2. Each line accounts for a combination of different ADVOS settings (blood flow/concentrate flow). As demonstrated for Supplementary Figure 3 and 4, for each of the settings, there is a correlation between measured and calculated values according to the Stewart approach. [file 40635_2019_269_MOESM4_ESM.zip › Figure S6.tif]

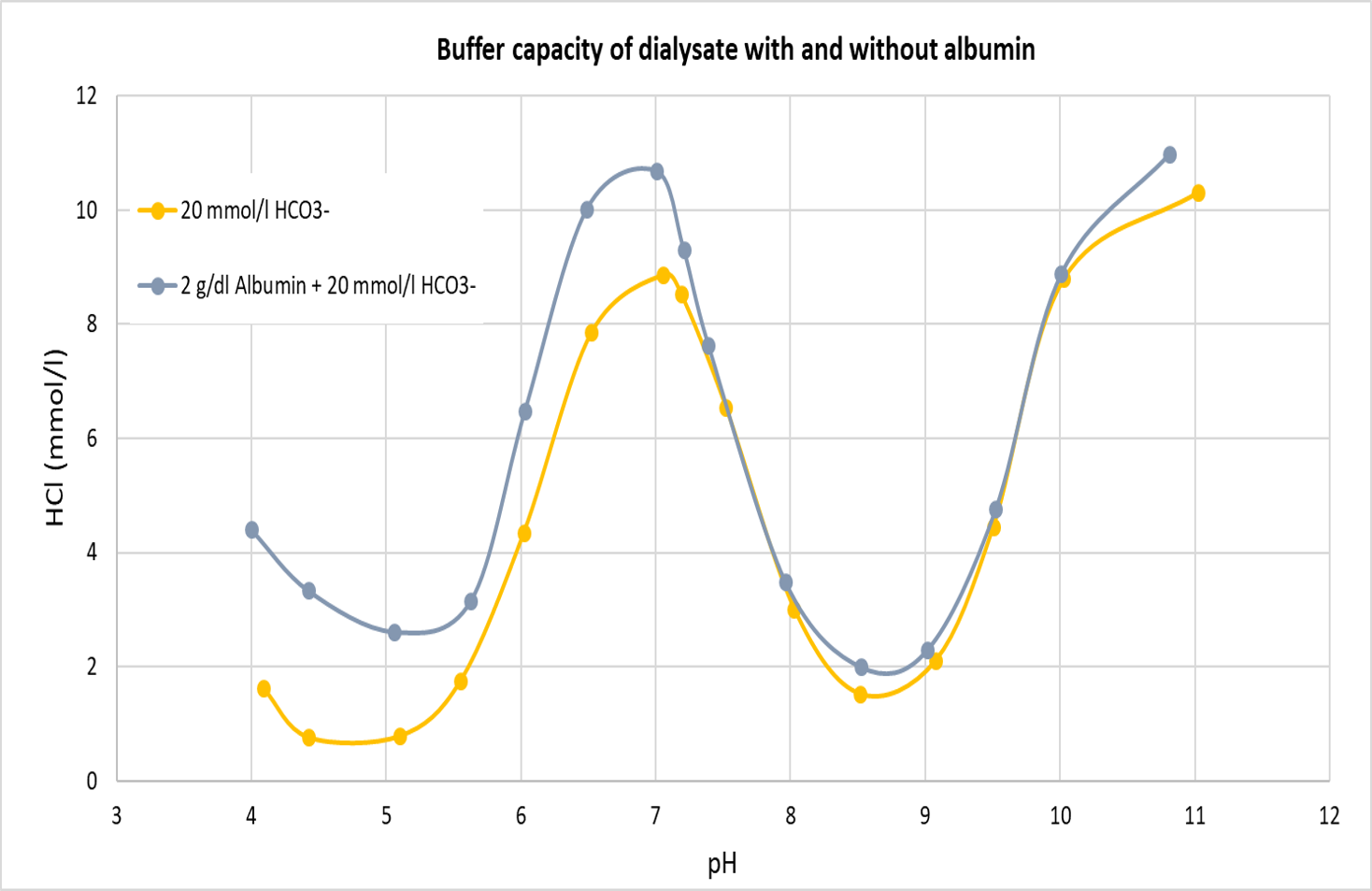

Supplement: Supplementary file 5 — Additional file 5: Figure S7. Buffer capacity of a dialysate containing 20 mmol/l sodium bicarbonate with or without albumin (2 g/dl). The buffer capacity (β) is defined as the moles of an acid or base necessary to change the pH of a solution by 1, divided by the pH change and the volume of buffer in liters. [file 40635_2019_269_MOESM5_ESM.tif]

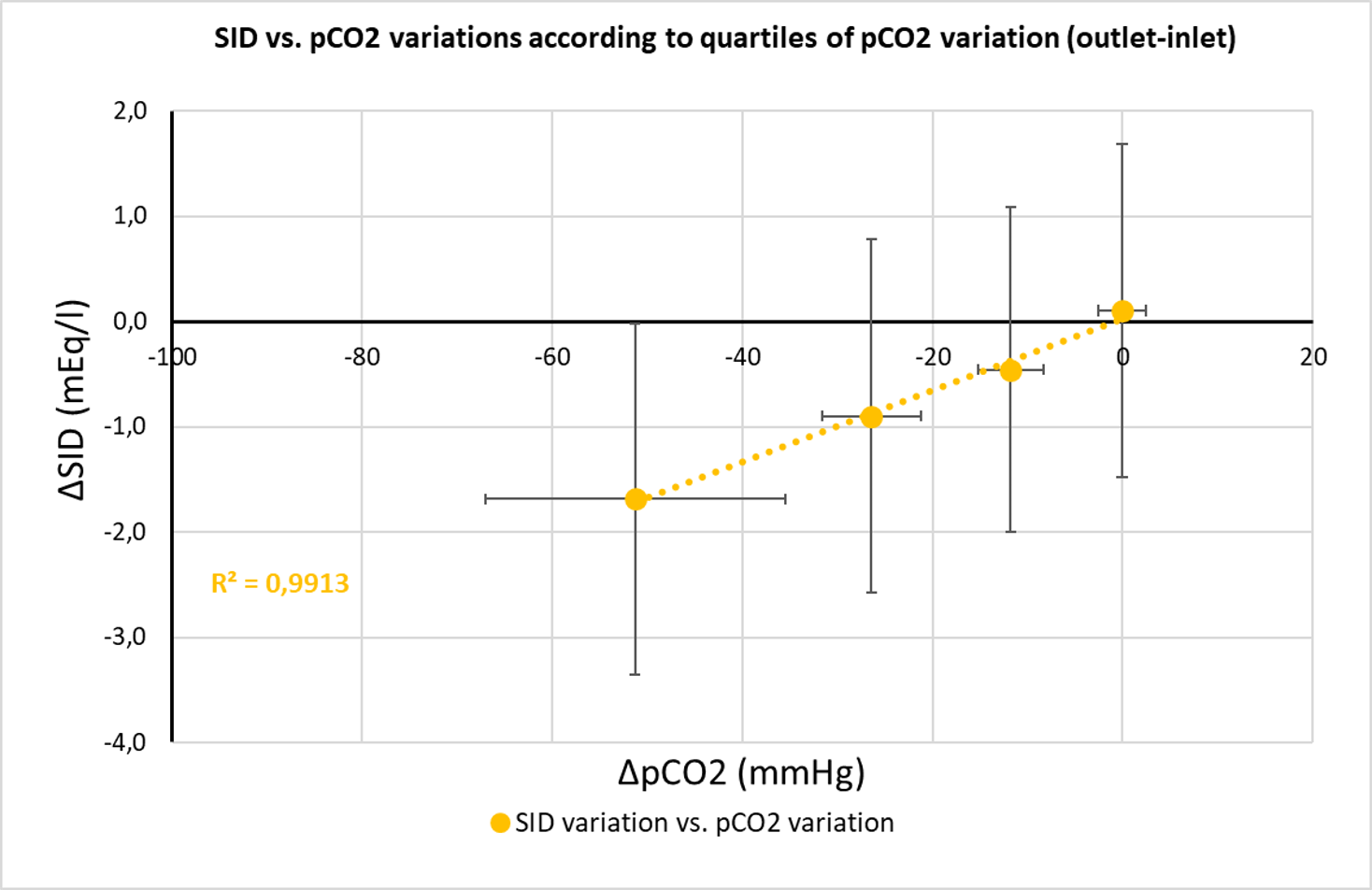

Supplement: Supplementary file 6 — Additional file 6: Figure S8. Analysis of SID variations (outlet – inlet) according to quartiles of pCO2 variation (outlet – inlet). As shown in [30]. Mean ± S.D. [file 40635_2019_269_MOESM6_ESM.tif]

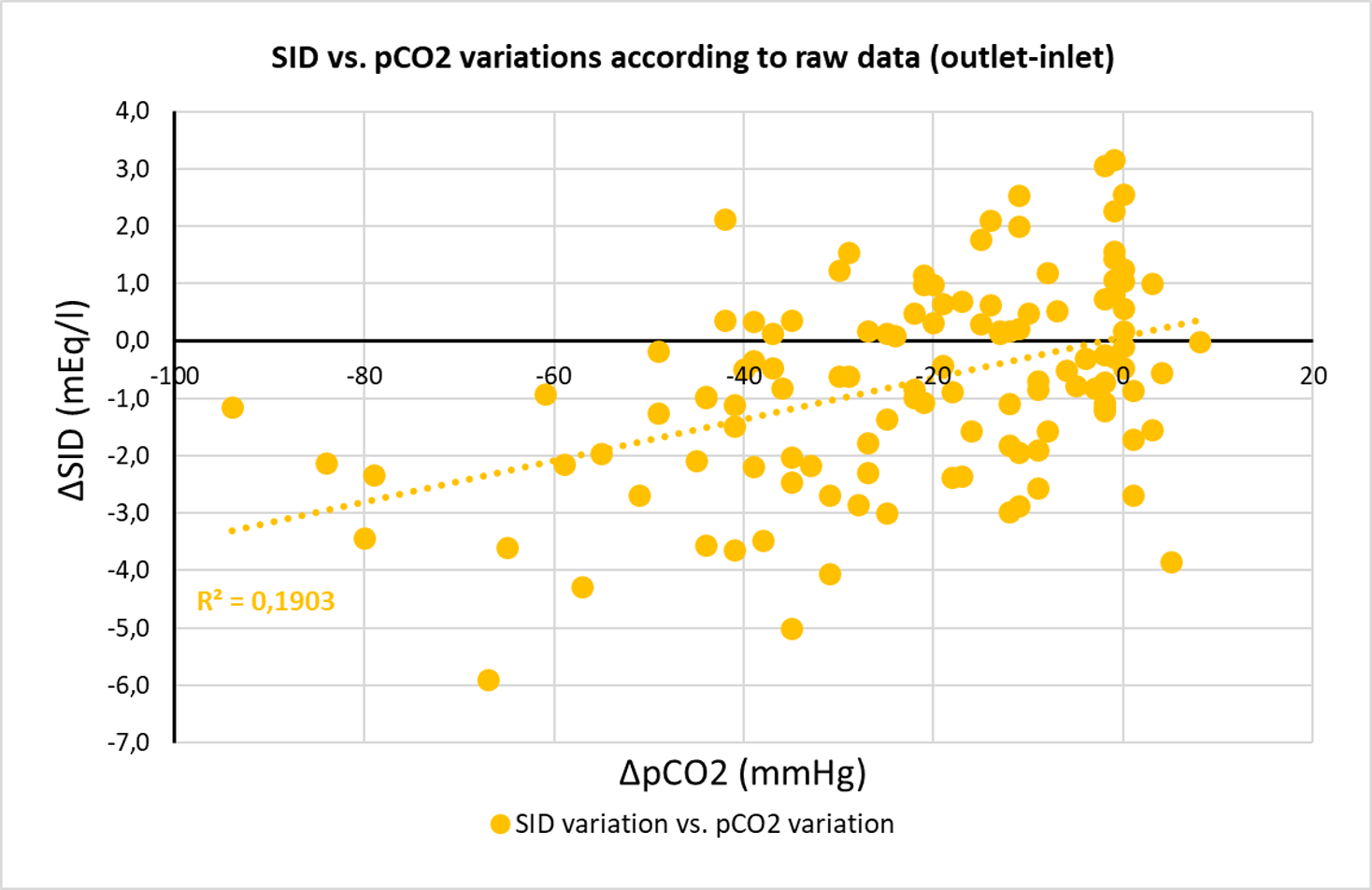

Supplement: Supplementary file 7 — Additional file 7: Figure S9. Correlation between SID variations (outlet – inlet) and pCO2 variation (outlet – inlet) using raw data. These data show, that in our experiments there is no interdependence between SID and pCO2 variation, contrary to what is described in [30]. Using quartiles for pCO2 variation as shown in Supp. Figure 8, an artefactual correlation might be created. [file 40635_2019_269_MOESM7_ESM.tif]
